# Modified Huang-Lian-Jie-Du Decoction Ameliorates A*β* Synaptotoxicity in a Murine Model of Alzheimer's Disease

**DOI:** 10.1155/2019/8340192

**Published:** 2019-11-03

**Authors:** Yan Liu, Ting Du, Wenlong Zhang, Weiye Lu, Zhichao Peng, Shuqiong Huang, Xiangdong Sun, Xiaoqin Zhu, Chaojun Chen, Linchao Qian, Lei Wen, Pingyi Xu, Yunlong Zhang

**Affiliations:** ^1^Key Laboratory of Neuroscience, School of Basic Medical Sciences, Guangzhou Medical University, Guangzhou 511436, China; ^2^Department of Traditional Chinese Medicine, School of Medicine, Xiamen University, Xiamen 361102, China; ^3^Department of Neurology, Guangzhou Chinese Medical Integrated Hospital (Huadu), Guangdong 510800, China; ^4^School of Basic Medical Sciences, Second Affiliated Hospital of Guangzhou Medical University, Guangzhou 510260, China; ^5^Guangzhou Medical University, Guangzhou 511436, China; ^6^Department of Neurology, The First Affiliated Hospital of Guangzhou Medical University, Guangzhou, China; ^7^Shenzhen Research Institute of Xiamen University, Shenzhen 518000, China

## Abstract

Alzheimer's disease (AD) is a common neurodegenerative disease, characterized by cognitive dysfunction; however, the therapeutic strategies are not fully understood. Huang-Lian-Jie-Du-Decoction (HLJDD) is a famous traditional Chinese herbal formula that has been widely used clinically to treat dementia. Recently, according to previous study and our clinical practice, we generate a new modification of HLJDD (named modified-HLJDD). In this study, we indicated that modified-HLJDD attenuated learning and memory deficiencies in A*β*_1-42_ oligomer-induced AD model, and we confirmed the exact metabolites in modified-HLJDD solution, as compared with HLJDD by UHPLC-Q-TOF-MS. Using GC-Q-TOF/MS-based metabolomics, we identified adenosine as the potential significant metabolite, responsible for modified-HLJDD regulating energy metabolism and synaptic plasticity in AD model. We also revealed that the potential underlying mechanism of modified-HLJDD in AD model may involve NMDA receptor-mediated glutamatergic transmission and adenosine/ATPase/AMPK cascade. Moreover, we also indicated the differential gut microbiota which mainly involved *Firmicutes*, *Bacteroidetes*, *Proteobacteria*, and *Actinobacteria* at the phylum level upon modified-HLJDD treatment in AD model. Based on the correlation of metabolomic analysis with microbiome analysis, we clarified that *Dorea* is the most affected microbiota with adenosine upon modified-HLJDD treatment in AD model. Thus, our study suggests that modified-HLJDD may serve as a potential therapeutic drug in treating AD.

## 1. Introduction

Alzheimer's disease (AD) is the most common neurodegenerative disorder, causing memory loss and cognitive dysfunction. Extracellular senile plaques and phosphorylated tau-associated intraneuronal neurofibrillary tangles (NFTs) are the two classical pathologic hallmarks in AD. Senile plaques comprise amyloid-*β* (A*β*), which is released from amyloid precursor protein (APP) after the sequential cleavages of APP by *β*- and *γ*-secretases. A*β*_1-40_ and A*β*_1-42_ are the most prevalent isoforms of A*β* oligomers in AD patients. A*β*_1-40_ is the most abundant, while A*β*_1-42_ has the capacity to form the core of A*β* plaque deposition before aggregation [1]. Oligomeric A*β* has been proven to disrupt glutamatergic receptor activity and impair long-term potentiation (LTP), a form of synaptic plasticity [2]. Synaptic loss is an early feature of AD, and it is closely correlated with the severity of dementia [3]. Oligomeric A*β* can induce synaptic loss via disruption of glutamatergic receptors, calcium homeostasis, and mitochondrial dynamics [4–6]. Thus, A*β* plays a crucial role in the etiology of AD, and A*β*-induced synaptic dysfunction mimics the early stages of AD pathogenesis [7]. However, the underlying mechanism of A*β*-induced synaptic collapse in the early stages of AD is still unclear.

Adenosine, an endogenous purinergic nucleoside, is a ubiquitous neuromodulator in the central nervous system. Adenosine can be generated from adenine nucleosides via 5′-nucleotidase enzymes intracellularly or extracellularly [8], and it regulates neuronal survival and neurotransmitter release of glutamate, aspartate, acetylcholine, and *γ*-aminobutyric acid [9–11]. Besides, adenosine has been proposed to be a neuroprotective agent against epilepsy, ischemia, AD, and Parkinson's disease (PD) [12–15]. These functions mainly involve the binding of adenosine to A_1_, A_2A_, A_2B_, and A_3_ receptors. The colocalization between adenosine A1 receptor (A1R) and A*β* in senile plaques has been found in the hippocampus of AD patients, and activation of A1R increases tau phosphorylation [16]. Additionally, a polymorphism of the *adenosine A2a receptor (ADORA2A)* gene has been reported to be associated with hippocampal volume in mild cognitive impairment and AD patients [17]. These results suggest that adenosine and its receptors are potential targets for AD. However, further studies are needed to better elucidate the role of adenosine in AD.

Although A*β* is believed to be highly associated with AD pathogenesis, to date, several immunotherapeutic strategies targeting A*β* have proven to be less clinically effective than had been anticipated [18, 19]. Nowadays, many components from herbs have been identified as effective in the treatment of neurodegenerative disease, such as AD and PD [20–23]. Huang-Lian-Jie-Du Decoction (HLJDD) is a famous traditional Chinese medicine (TCM) formula widely used in treating stroke, inflammation, and dementia in the Far East. It consists of four herbs—Rhizoma Coptidis (*Coptis chinensis* Franch., Ranunculaceae), Radix Scutellariae (*Scutellaria baicalensis* Georgi., Lamiaceae), Cortex Phellodendri (*Phellodendron amurense* Rupr., Rutaceae), and Fructus Gardeniae (*Gardenia jasminoides* Ellis., Rubiaceae)—with the dry-weight ratio of 3 : 2 : 2 : 3. Previously, HLJDD extracts—its modified formula, as well as its major components—have been proven to prevent learning and memory deficits in cell culture and animal models of AD [24–26]. Durairajan et al. reported that Radix Scutellariae can enhance A*β* generation by increasing the protein level of APP, and they detected neuroprotective effects of HLJDD without Radix Scutellariae [24]. Moreover, in a Japanese case report, the addition of orengedoku-to (the same prescription of HLJDD in Japan) to yokukan-san (Kampo prescription) exerted the same efficacy as aripiprazole in controlling aggressiveness in an Alzheimer's-type dementia patient without any adverse effects [27]. However, the exact mechanism underlying HLJDD-mediated cognitive improvements in AD is still unknown.

In this study, we examined the neuroprotective effects of a newly modified-HLJDD (also named the Jie-Du-Hua-Yu Decoction)—which is HLJDD without Radix Scutellariae, but with the addition of Salvia miltiorrhiza, Curcuma longa L., and Acorus tatarinowii—on an A*β*-induced AD mouse model. Here, we report that modified-HLJDD ameliorated learning and memory deficiency in our AD mouse model. Furthermore, using gas chromatography quadrupole time of flight mass spectrometry- (GC-Q-TOF/MS-) based metabolomics and the 16S-rDNA microbiome, we revealed their correlations following application of modified-HLJDD in our AD mouse model. Modified-HLJDD improving cognitive behavior may be correlated with the alteration of metabolites and gut-microbial compositions via regulating NMDA receptors and adenosine signaling. Thus, modified-HLJDD may serve as a potential agent in treating cognitive impairment in AD.

## 2. Materials and Methods

### 2.1. Reagents

Rhizoma Coptidis, Radix Scutellariae, Cortex Phellodendri, Fructus Gardeniae, Salvia miltiorrhiza, Acorus tatarinowii, and Curcuma longa L. were purchased from Xiamen Yanlaifu Pharmaceutical Co., Ltd. (Xiamen, China). Amyloid-*β* (1–42) peptide was purchased from AnaSpec (AS-20276, San Jose, CA, USA). Anti-phospho-AMPK (Thr172) (#2535, 1 : 1000), NMDAR1 (#5704, 1 : 1000), NMDAR2A (#4205, 1 : 1000), NMDAR2B (#4212, 1 : 1000), GluA1 (#13185, 1 : 1000), GluA2 (#13607, 1 : 1000), synapsin (#5297, 1 : 1000), synaptotagmin (#14558, 1 : 1000), syntaxin (#18572, 1 : 1000), PSD-95 (#3450, 1 : 1000), and GAPDH (#5174, 1 : 1000) antibodies were purchased from Cell Signaling Technology (Danvers, MA, USA). Anti-NeuN (MAB377, 1 : 100) and glial fibrillary acidic protein (GFAP) (MAB360, 1 : 400) antibodies were purchased from Millipore (Bedford, MA, USA). Anti-Iba1 (019-19741, 1 : 500) antibody was purchased from Wako (Chuo-Ku, Osaka, Japan). Anti-phospho-ATPase (Ser16) (E1A3C83, 1 : 1000), ATPase (E1A6083, 1 : 1000), and AMPK (E1A6423, 1 : 1000) antibodies were purchased from EnoGene Biotechnology (Nanjing, China). Anti-Alexa Fluor 488-conjugated goat anti-mouse, anti-Alexa Fluor 594-conjugated goat anti-rabbit, horseradish peroxidase-conjugated goat anti-mouse, and rabbit antibodies were purchased from Boster (Wuhan, China). Fatty acid methyl ester (C7-C30, FAMEs) standards, methoxyamine HCl, pyridine, and anhydrous sodium sulfate were purchased from Sigma-Aldrich (St. Louis, MO, USA). MSTFA (N-methyl-N-(trimethylsilyl)trifluoroacetamide) with 1% (vol/vol) trimethylchlorosilane (MSTFA, with 1% TMCS), methanol (Optima LC-MS), acetonitrile (Optima LC-MS), hexane, dichloromethane, chloroform, and acetone were purchased from Thermo-Fisher Scientific (Fair Lawn, NJ, USA). Ultrapure water was produced by a Milli-Q Reference system equipped with a LC-MS Pak filter (Millipore, Bedford, MA, USA).

### 2.2. Animals

Eight-week-old male C57BL/6 mice were obtained from SLAC Laboratory Animal Co., Ltd. (Shanghai, China). Three mice per cage had free access to food and water and were housed with a 12 : 12 h light/dark cycle, with lights on from 06:00 to 18:00, and the facility was maintained at a constant temperature and humidity. Mice were allowed to adapt to the environment for at least one week before experiments. All the experiments were conducted according to the National Institute of Health guidelines on the care and use of animals (NIH Publications No. 8023, revised 1978) and approved by the Institutional Animal Care and Use Committee of Guangzhou Medical University.

### 2.3. Preparation of HLJDD and Modified-HLJDD

The TCM formula for HLJDD in our study was composed of four herbs, namely, Rhizoma Coptidis, Radix Scutellariae, Cortex Phellodendri, and Fructus Gardeniae. In contrast, modified-HLJDD was composed of six herbs, namely, Rhizoma Coptidis, Cortex Phellodendri, Fructus Gardeniae, Salvia miltiorrhiza, Acorus tatarinowii, and Curcuma longa L. The TCM formula was prepared in reference to the optimized method described by Chen et al. [28]. HLJDD (Rhizoma Coptidis, Radix Scutellariae, Cortex Phellodendri, and Fructus Gardeniae) was crushed into small pieces and mixed in a ratio of 3 : 2 : 2 : 3 (weight), and the modified-HLJDD (Rhizoma Coptidis, Cortex Phellodendri, Fructus Gardeniae, Salvia miltiorrhiza, Curcuma longa L., and Acorus tatarinowii) was mixed in a ratio of 3 : 2 : 3 : 3 : 2 : 2 (weight). The mixture was refluxed with water (1 : 10, *w*/*v*) for 2 h, filtrates were collected, and the residues were then refluxed in water (1 : 10, *w*/*v*) for 1.5 h. The extract solutions were combined and were concentrated to 0.875 g/ml (for HLJDD) and 1.312 g/ml (for modified-HLJDD).

### 2.4. A*β* Oligomer Preparation and Injection

In this study, we aimed to examine the early stages of A*β* oligomer damage via local administration of A*β* peptide into the mouse brain. A*β*_1–42_ peptides were dissolved in hexafluoroisopropanol (HFIP) to 1 mg/ml and incubated at room temperature for 2 h to allow for A*β* monomerization. Then, the A*β*_1–42_ film was resuspended by adding DMSO, which was then sonicated and stored at −20°C until further use. A*β* oligomer aggregation was performed as described by Fa et al. [29]. Briefly, A*β*_1–42_ stock solution was diluted into phosphate-buffered saline (PBS) at 200 *μ*M and incubated at 4°C for 48 h to enhance oligomer formation. Transmission electron microscopy was used to examine the morphology of aggregated A*β*_1–42_ forms.

Two microliters of A*β*_1-42_ oligomer (200 *μ*M) was injected i.c.v. as described previously. Briefly, mice were anesthetized (2% isoflurane in 1 l/min oxygen/nitrous oxide) and placed in a stereotaxic frame, and the A*β*_1-42_ oligomer was delivered into the right side of the lateral ventricle at the target site (bregma AP, −0.2 mm; ML, +1.2 mm; and DV, −2.0 mm). A Hamilton syringe was filled with A*β*_1-42_ oligomer, and the needle was lowered into the tissue at a rate of 0.5 *μ*l/min. The syringe was left in place for 5 min before being slowly withdrawn from the brain, after which the wound was cleaned and sutured. Control mice received the equivalent volume of PBS into the lateral ventricle.

### 2.5. Animal Experiment

The C57BL/6 mice were divided into six groups (*n* = 12 each group): control, intracerebroventricular injection of A*β*_1–42_, HLJDD plus A*β*_1–42_, low dose of modified-HLJDD plus A*β*_1–42_, high dose of modified-HLJDD plus A*β*_1–42_, and donepezil plus A*β*_1–42_. HLJDD and modified-HLJDD were prepared as mentioned above. Mice in a low dose of modified-HLJDD groups were intragastrically administered at a final concentration of 3.5 g·kg^−1^, while mice in HLJDD and a high dose of modified-HLJDD group were intragastrically administered at a final concentration of 7 g·kg^−1^ (according to the ratio in the raw-medicinal material). Donepezil was set as the positive control, and mice in this group were intragastrically administered at a final concentration of 2 mg·kg^−1^. Mice in the control and intracerebroventricular injection of A*β*_1–42_ groups were intragastrically administered with sterilized distilled water. Three days after intracerebroventricular injection of A*β*_1–42_, mice were intragastrically given drug or distilled water each day for 3 weeks. One day after the last drug/water administration, behavioral tests were performed.

### 2.6. Metabolomic-Based Analysis of HLJDD and Modified-HLJDD Solutions by Ultraperformance Liquid Chromatography Quadrupole Time of Flight Mass Spectrometry (UPLC-Q-TOF/MS)

HLJDD and modified-HLJDD solutions were extracted with 1000 *μ*l of extraction liquid (*V* methanol : *V* acetonitrile = 1 : 1), and then, 150 *μ*l of water was added and the solutions were vortexed for 30 s. The extractions were homogenized in a ball mill for 4 min at 45 Hz and then treated with ultrasound for 5 min. The homogenization was repeated three times and then incubated for 1 h at -20°C to precipitate proteins. After centrifugation at 12000 rpm for 15 min at 4°C, the supernatant was transferred into a new Eppendorf tube. The extracts were dried in a vacuum concentrator, and 200 *μ*l of extraction liquid was added (*V* acetonitrile : *V* water = 1 : 1) to the resuspension. The suspension was then vortexed for 30 s, sonicated for 10 min, and centrifuged for 15 min at 12000 rpm at 4°C. The supernatant was transferred into a fresh 2 ml LC/MS glass vial for the UHPLC-Q-TOF-MS analysis.

LC-MS/MS analyses were performed using a UHPLC system (1290, Agilent Technologies, Palo Alto, CA, USA) with a UPLC BEH Amide column (2.1 × 100 mm^2^, 1.7 *μ*m, Waters Corp., Milford, USA) coupled to TripleTOF 5600 (Q-TOF, AB Sciex). The mobile phase consisting of 25 mM NH_4_OAc and 25 mM NH_4_OH in water (pH = 9.75) (A) and acetonitrile (B) was carried out with an elution gradient as follows: 0 min, 95% B; 7 min, 65% B; 9 min, 40% B; 9.1 min, 95% B; and 12 min, 95% B, which was delivered at 0.5 ml/min. The TripleTOF mass spectrometer was used for its ability to acquire MS/MS spectra on an information-dependent basis (IDA) during an LC/MS experiment. In this mode, the acquisition software (Analyst TF 1.7, AB Sciex) continuously evaluates the full-scan survey MS data as it collects and triggers the acquisition of MS/MS spectra depending on preselected criteria. In each cycle, 12 precursor ions with an intensity greater than 100 were chosen for fragmentation at a collision energy (CE) of 30 V (15 MS/MS events with product ion-accumulation time of 50 ms each). ESI (electron spray ionization) source conditions were set as the following: ion-source gas 1 as 60 psi, ion-source gas 2 as 60 psi, curtain gas as 35 psi, source temperature of 650°C, and ion spray voltage floating (ISVF) of 5000 V or -4000 V in positive or negative modes, respectively. The MS raw data (.wiff) files were converted to the mzXML format using ProteoWizard and processed by R package XCMS (version 3.2). The preprocessing results generated a data matrix that consisted of the retention time (s), mass-to-charge ratio (*m*/*z*) values, and peak intensity. An R-package CAMERA was used for peak annotation after XCMS data processing. An in-house MS2 database was applied for metabolite identification.

### 2.7. Behavioral Tests

Behavioral tests were performed on the day after the last drug/water administration.

#### 2.7.1. Open Field Test (OFT)

Mice were gently placed in the center of an open field arena (50 cm (*L*) × 50 cm (*W*) × 40 cm (*H*)) for 5 min. The arena was brightly illuminated and had a central zone (25 cm × 25 cm) and a peripheral zone. During the experiments, the open field was video recorded. We measured total travel distance and time spent in the center and peripheral zones of the maze via the Smart 3.0 video tracking system (Panlab, Barcelona, Spain). After each trial, the apparatus was cleaned with 75% ethanol.

#### 2.7.2. T-/Y-Maze

The T-/Y-maze was used for evaluation of spontaneous alternation for spatial working memory in mice. Briefly, one of the arms was blocked with a plastic sliding door (defined as the novel arm), and the mouse was allowed to enter and explore the two open arms for 5 min. Thirty minutes later, the sliding door was removed, and the mouse was returned to the maze and allowed to freely enter any of the three open arms for another 5 min. The series of arm-entries and the time spent in each arm were calculated manually from a video recording made with Smart 3.0 video tracking software. The percentage of time spent in the novel arm was calculated as the ratio of time spent in the novel arm out of the total time spent in all three arms for each group. The maze was thoroughly cleaned with 75% ethanol between tests with different animals.

#### 2.7.3. Morris Water Maze Test

The Morris water maze (MWM) test was performed as stated previously [30]. MWM consisted of a pool (diameter: 120 cm) filled with water (22 ± 1°C), which was made opaque-white with bright white food coloring. An invisible platform (10 cm^2^) that was submerged 2 cm beneath the water surface was placed in the center of one of the four quadrants of the pool (NE, SE, SW, and NW), and different images (circles, squares, and triangles) serving as reference cues were hung on the pool walls. Training was conducted over five consecutive days, with four trials per day, using an intertrial interval of 1–1.5 min. Mice were released in the water in one of the four quadrants randomly. In each trial, mice swam until they found the hidden platform or were gently guided to it by the trainer if not found within 60 s. Mice remained on the platform for 15 s before being returned to the home cage. Daily data were averaged across the four trials. On day six, a probe trial was conducted—in which the hidden platform was removed—and mice were placed in the pool and allowed to swim for 60 s. The time of crossing through the original platform position, the time spent in the target quadrant, and the swimming speed were monitored by a camera. Images and swimming paths were stored in a computer and analyzed automatically using Smart 3.0 video tracking software (Panlab, Barcelona, Spain).

### 2.8. Tissue Preparation

Mice in each group were euthanized using isoflurane, and tissues were collected for further analysis utilizing various assays: (1) Western blotting and adenosine level test assays: mice were anesthetized and perfused transcardially with 0.9% saline to remove traces of blood. Hippocampal tissues were collected and stored at −80°C. (2) Morphological experiments (immunofluorescence and immunohistochemistry): Mice were anesthetized and perfused transaortally with 0.9% saline followed by fixative (4% paraformaldehyde in 0.01 M PBS, pH 7.4). The fixed brains were collected, stored in postfix solution (4% paraformaldehyde in 0.01 M PBS, pH 7.4) overnight at 4°C, and dehydrated in a gradient of 20–30% sucrose. Then, the embedded brains were cut into sections of 15 *μ*m with a freezing microtome (Leica, Germany) and subsequently stored at −80°C until use. (3) Metabolomic analysis assay: hippocampal tissues were collected and subjected to metabolomic analysis as mentioned below. (4) 16S rDNA analysis assay: feces samples were collected (from 8:00 am to 16:00 pm) using metabolic cages with ice-packed Eppendorf tubes and immediately stored at −80°C until analysis.

### 2.9. Western Blotting Assay

As stated previously [31], prepared samples were subjected to gel electrophoresis (12% SDS-PAGE) and probed using relevant antibodies. Peroxidase activity was examined by enhanced chemiluminescence (Millipore, MA, USA), and chemiluminescent immunoreactive complexes were collected using the Tanon imaging system (Shanghai, China). Protein levels were quantified using ImageJ software. GAPDH immunoreactivity was set as the control.

### 2.10. Immunofluorescent Assay

The immunofluorescence assay was performed as described previously [23, 30, 32]. For immunostaining, the prepared brain slices were incubated with primary antibodies (NeuN and synapsin, NeuN and Iba1) overnight at 4°C, rinsed with PBS, and incubated with Alexa Fluor 488-conjugated goat anti-mouse and Alexa Fluor 594-conjugated goat anti-rabbit IgG for 2 h at 37°C. DAPI was used to stain cell nuclei. Immunostaining was then examined using an Olympus FV1000-1X81 laser-scanning confocal microscope (Shinjuku, Tokyo, Japan).

### 2.11. Immunohistochemical Assay

Immunohistochemical assay was performed as described previously [23, 30, 32]. For immunohistochemistry of brain sections, we performed antigen retrieval by treating the mounted cryostat sections with citrate buffer at 95°C for 15 min followed by cooling to room temperature for 1 h. Sections were rinsed in PBS and blocked with 5% BSA for 1 h at 37°C. The slices were incubated with primary antibodies (p-AMPK and GFAP) overnight at 4°C. After washing with PBS, the slices were incubated with secondary antibodies for 2 h at 37°C. Antibody-peroxidase complexes were revealed by incubating the slices with 3,3-diaminobenzidine peroxidase substrate (Boster, Wuhan, China). Hippocampal p-AMPK and GFAP immunostaining were determined by the Image-Pro Plus 6.0 photogram analysis system (IPP 6.0, Media Cybernetics, Bethesda, MD, USA).

### 2.12. Adenosine Level Test

One day after the behavioral tests, hippocampal samples were collected and subjected to test the adenosine level. Hippocampal samples were lysed with RIPA with protein inhibitors and adenosine deminase inhibitor EHNA hydrochloride (E114, Sigma, St. Louis, MO, USA). Then, hippocampal adenosine is measured using the Adenosine Assay Kit (K327-100, BioVision, Milpitas, CA, USA). Fluorescence was measured using a multimode plate reader (VICTOR Nivo, PerkinElmer, Waltham, MA, USA). Adenosine in samples was calculated based on a calibration curve from standard adenosine samples, and the hippocampal adenosine level was expressed as 100% of the control group.

### 2.13. Metabolomic Analysis of Hippocampal Samples

The untargeted metabolomic profiling was performed on the XploreMET platform (Metabo-Profile, Shanghai, China). Chemicals and reagents used in the metabolomic analysis are listed above, and the details of their use are listed below.

#### 2.13.1. Sample Preparation

The frozen hippocampal samples harvested from these four groups (control, model, HLJDD, and modified-HLJDD) were mixed with 25 mg of prechilled zirconium oxide beads, 10 *μ*l of internal standard, and 50 *μ*l of 50% prechilled methanol. After centrifugation at 14000 g at 4°C for 20 min, the supernatant was then transferred to an autosampler vial (Agilent Technologies, Foster City, CA, USA). The residue was added to prechilled methanol/chloroform (*v*/*v* = 3/1) for the second extraction. The mixtures in the autosampler vial were evaporated to remove chloroform and lyophilized with a FreeZone freeze dryer (Labconco, Kansas City, MO, USA). Then, the dried sample was derivatized with methoxyamine (20 mg/ml in pyridine) at 30°C for 2 h, followed by addition of MSTFA (1% TMCS), containing FAMEs as retention indices, at 37.5°C for another 1 h. Then, the derivatized samples were injected to GC-TOF/MS for metabolomic analysis.

To evaluate reproducibility and stability of the GC-TOF/MS analysis system, randomly selected cell-pellet samples from each group were mixed to generate a pooled quality-control sample (pooled QC samples). The QC samples were injected at the beginning and end and at regular intervals (after every 12 test samples) throughout the analytical run.

#### 2.13.2. Analysis Conditions

Chromatographic experiments were performed on a Rxi-5ms capillary column (30 m × 250 *μ*m; i.d., 0.25 *μ*m film thickness (Restek Corporation, Bellefonte, PA, USA)). Helium was used as the carrier gas at a constant flow rate of 1.0 ml/min. The temperature of injection and transfer interface were both set to 270°C. The source temperature was 220°C. The measurements were made using electron-impact ionization (70 eV) in the full-scan mode (*m*/*z*). MS analysis was performed on a GC-TOF (Pegasus HT, LECO Corp., St. Joseph, MO, USA). The instrument was operated by using an electrospray-ionization source in the positive mode. The ionization source conditions were as follows: detector voltage, 1450 V; source temperature, 220°C; acquisition rate, 25 spectra/s; and mass range, 50-500 Da.

#### 2.13.3. Pattern Recognition Analysis and Data Processing

Metabolite annotation was performed by comparing the retention indices and mass spectral data with the JiaLib metabolite database (purchased from Sigma-Aldrich (St. Louis, MO, USA), Santa Cruz (Dallas, TX, USA), and Nu-Chek Prep (Elysian, MN, USA)). The raw data generated by GC-TOF/MS were processed using XploreMET (v3.0, Metabo-Profile, Shanghai, China) for automated-baseline denoising and smoothing, peak picking and deconvolution, creating a reference database from the pooled QC samples, metabolite signal alignment, missing value correction and imputation, and QC correction. The resultant data matrices were subsequently introduced into XploreMET software (v3.0, Metabo-Profile, Shanghai, China) for principal component analysis (PCA), partial least-square discriminant analysis (PLS-DA), and orthogonal partial least-square discriminant analysis (OPLS-DA). Each dataset was transformed into comparable data vectors for statistical analysis. Prior to PCA, PLS-DA, and OPLS-DA, all measurements were mean-centered and scaled by the standard deviation of the observed measurements. The PLS-DA score plots were described by the cross-validation parameters R2Y and Q2, which represent the total explained variation for the *X* matrix and the predictability of the model, respectively. The value of variable importance in the projection (VIP) was used to weight the sum of squares of the PLS weights, reflecting the relative contribution of each *X* variable to the model. Those variables with VIP > 1.0 were considered significantly different between classes. SPSS 16.0 software was used for statistical calculations; comparisons between two groups were performed by Student's *t*-test, and comparisons among four groups were performed by one-way analysis of variance (ANOVA). The significant standard-boundary value was set to *p* < 0.05. MetaboAnalyst software was used for the pathway analysis; this software is a web-based tool for visualization of metabolomics, as reported previously [33].

### 2.14. 16S rDNA Analysis of Fecal Samples

The 32 fecal samples from these four groups were collected immediately after behavioral tests. Samples were placed in 1.5 ml tubes, snap-frozen on dry ice, and stored at −80°C. The 16S rDNA analysis of the fecal samples was performed by Anjie Medical Co., Ltd. (Xiamen, China). DNA extraction was performed using PowerFecal DNA kits (QIAGEN, Duesseldorf, Germany). Genomic DNA was amplified in 50 *μ*l triplicate reactions with bacterial 16S rDNA gene (V3–V4 regions)-specific primers: 341F (5′-CCTAYGGGRBGCASCAG-3′) and 806R (5′-GGACTACNNGGGTATCTAAT-3′). The reverse primer contained a sample barcode, and both primers were connected with an Illumina sequencing adapter. PCR products were purified, and the concentrations were adjusted for sequencing on an Illumina HiSeq2500 system. Original sequencing reads from the sample were sorted by unique barcodes, followed by the removal of the barcode, linker, and PCR primer sequences. The resultant sequences were screened for quality, and ≥70 base pairs were selected for bioinformatic analysis. All sequences were classified using the BLAST and SILVA databases. After filtering the raw reads, all the remaining reads were assigned to operational taxonomic units (OTUs) with a 97% threshold of distance-based similarity and classified according to the QIIME reference database. Summaries of the taxonomic distributions of OTUs were constructed with these taxonomies and were used to calculate the relative abundances of microbiota at different taxonomic levels. Separately, distance calculation, operational taxonomic unit cluster, rarefaction analysis, and estimator calculation (*α*-diversity and *β*-diversity) were performed by the mothur program.

### 2.15. Spearman Correlation Analysis

To assess the relationship between microbiome and metabolome, the Mann-Whitney-Wilcoxon test and Student's *t*-test were firstly used to identify the significantly differential OTUs and metabolites, respectively. Subsequently, Spearman correlation coefficients between significant OTUs and metabolites were calculated. Correlations were evaluated at the phylum and genus levels. Heatmap, circos, and network topology were applied for visual display. All statistical analyses were performed in R-3.5.3 (https://www.r-project.org/).

### 2.16. Statistics

Statistical analysis of the data was performed in SPSS 16.0 (SPSS Inc., Chicago, IL, USA) using ANOVA followed by the Bonferroni *post hoc* test for multiple comparisons and Student's *t*-test for comparisons between two groups. All data are expressed as the mean ± standard error of the mean (SEM), and the statistical significance level was set at *p* < 0.05.

## 3. Results

### 3.1. Modified-HLJDD Attenuates Memory Deficiency in A*β*_1-42_ Oligomer-Treated Mice

**Figure 1:**
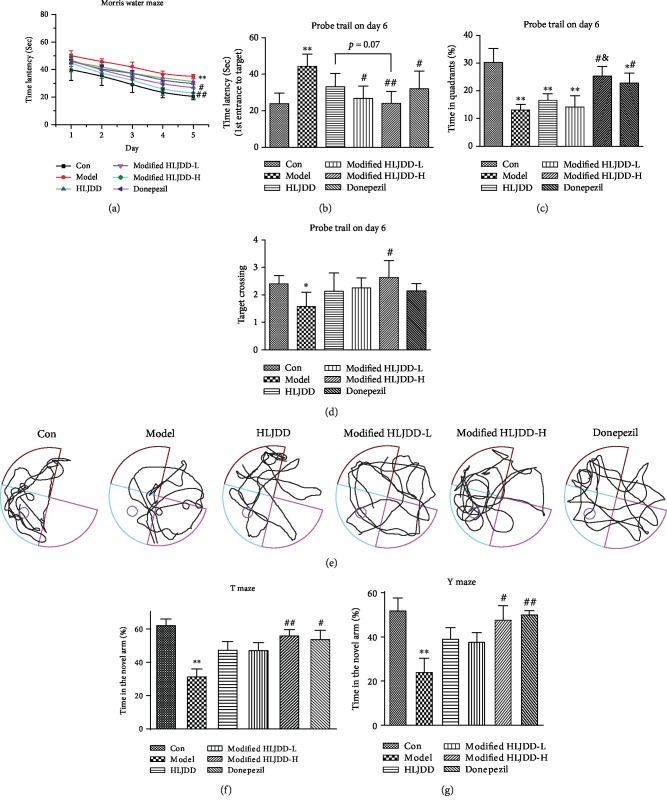
Modified-HLJDD attenuates learning and memory impairment in the A*β*_1-42_ oligomer-induced AD mouse model. (a–e) MWM tests were conducted after treatment with HLJDD, modified-HLJDD-L, modified-HLJDD-H, or donepezil. (a) The escape latency during a five-day training course. (b) In the probe tests, mice were analyzed for the escape latency; (c) the time spent in the target zone; (d) target crossing. (e) Representative path tracings in each quadrant during the probe trial. (f, g) T-maze/Y-maze results after HLJDD, modified-HLJDD-L, modified-HLJDD-H, or donepezil treatment in the AD model are presented as time spent in the novel arm. *n* = 12 per group. Results are expressed as the mean ± SEM. ^∗∗^*p* < 0.01, ^∗^*p* < 0.05 vs. control group; ^##^*p* < 0.01, ^#^*p* < 0.05 vs. model group; and ^&^*p* < 0.05 vs. HLJDD group. Statistical significance was determined by one-way ANOVA and Bonferroni tests as *post hoc* comparisons.

To investigate the effects of modified-HLJDD on the memory deficiency of A*β*_1-42_ oligomer-treated mice, we had mice perform the MWM, T-maze, and Y-maze. In the MWM, A*β*_1-42_ oligomer-treated mice showed impaired learning ability with increased escape latency during the five-day training phase and the probe trial test and with decreased time in the target zone and decreased target crossing (Figures 1(a)–1(e)). Moreover, A*β*_1-42_ oligomer-treated mice also showed decreased exploratory time in the T-maze and Y-maze (Figures 1(f) and 1(g)). These results suggest that A*β*_1-42_ oligomer-treated mice are suitable models for examining the effects of modified-HLJDD on memory impairment. Next, a high dose of modified-HLJDD and donepezil treatments decreased the escape latency during the five-day training phase and the probe trial test in AD model, while a low dose of modified-HLJDD treatment decreased the escape latency in the sixth day's probe trial test in AD model (Figures 1(a) and 1(b)). Furthermore, modified-HLJDD and donepezil treatment increased the time in the target zone in A*β*_1-42_ oligomer-treated mice, and modified-HLJDD showed much greater efficacy compared to that of HLJDD (Figure 1(c)). Only modified-HLJDD treatment promoted target crossing in A*β*_1-42_ oligomer-treated mice (Figure 1(d)). The swim speed showed no significant difference among these groups (Supplementary Fig. [Supplementary-material supplementary-material-1] and [Supplementary-material supplementary-material-1]). Representative path tracings in each quadrant during the probe trial are shown in Figure 1(e). In the T-/Y-maze test, both high dose of modified-HLJDD and donepezil treatments increased the exploratory time in the T-/Y-maze in A*β*_1-42_ oligomer-treated mice (Figures 1(f) and 1(g)). In addition, we also performed the open field test to examine general locomotor-activity levels, anxiety, and willingness to explore in A*β*_1-42_ oligomer-treated mice. Intriguingly, we found that A*β*_1-42_ oligomer injection induced mice to spend less time in the center and more time in the periphery of the open field compared with that of the control group (Supplementary Fig. [Supplementary-material supplementary-material-1]-[Supplementary-material supplementary-material-1]). Both HLJDD and donepezil treatment improved the performance of A*β*_1-42_ oligomer-treated mice in the open field test, and HLJDD was much more efficacious than modified-HLJDD in the open field test (Supplementary Fig. [Supplementary-material supplementary-material-1]-[Supplementary-material supplementary-material-1]). These results indicate that modified-HLJDD is more effective in attenuating memory deficiency in the A*β*_1-42_ oligomer-treated AD model.

### 3.2. Identification and Validation of Significant Metabolites between HLJDD and Modified-HLJDD Extract Solutions through Global Metabolomics

**Figure 2:**
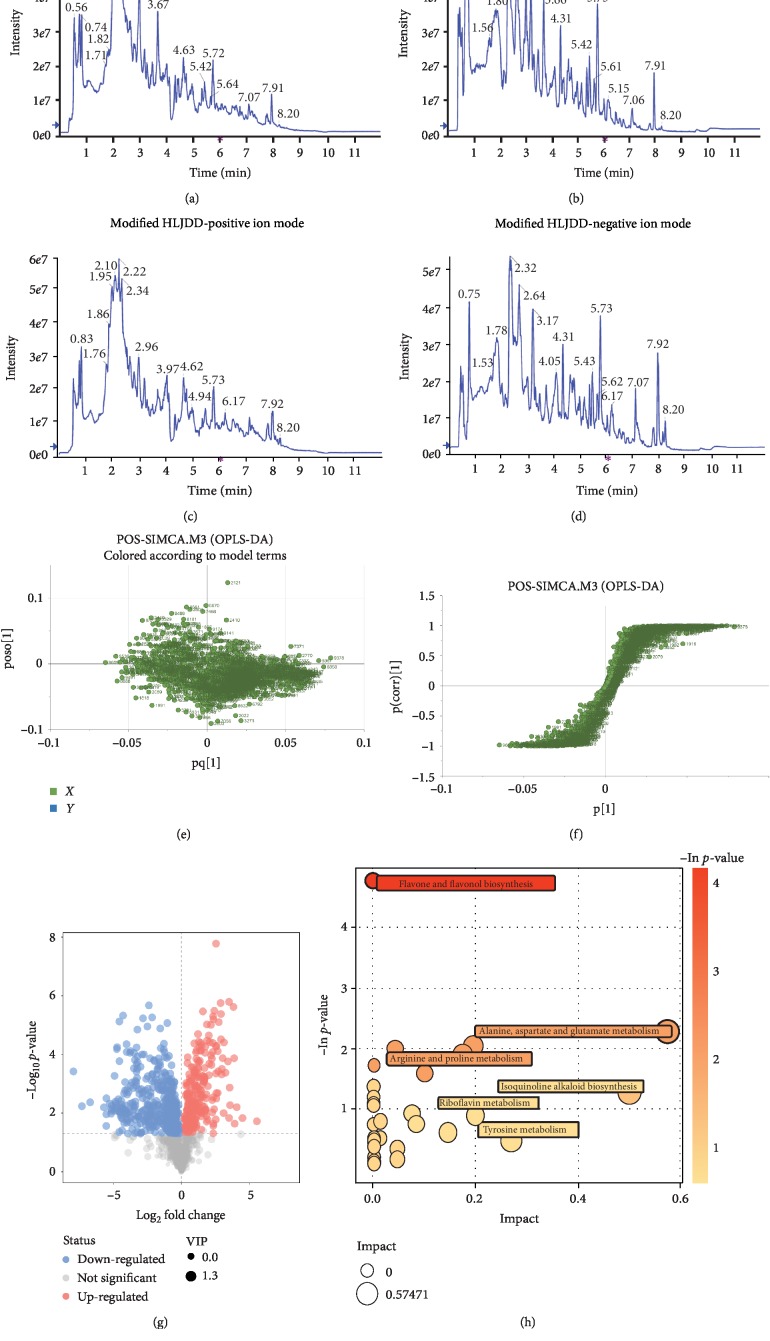
Identification of significant metabolites between HLJDD and modified-HLJDD extract solutions through UPLC-Q-TOF/MS. (a–d) The TCI of HLJDD and modified-HLJDD solutions under positive and negative ion modes. (e, f) Loading plot of PLS-DA and S-plot of OPLS-DA for HLJDD and modified-HLJDD. (g) The volcano plot showing all the significantly changed metabolites between HLJDD and modified-HLJDD solutions. (h) Pathway analysis for HLJDD and modified-HLJDD shown as a bubble plot. All assays were performed in triplicate.

Modified-HLJDD was derived from HLJDD, and modified-HLJDD was more effective in attenuating memory deficiency in our AD model. In the following experiments, we mainly focus on the effects of modified-HLJDD and HLJDD in A*β*_1-42_ oligomer-treated mouse model. Firstly, we aimed to explore the novel metabolites induced from modification of HLJDD. The HLJDD and modified-HLJDD solutions were analyzed by UPLC-Q-TOF/MS in the positive and negative ion modes. The total ion chromatograms (TCI) of HLJDD and modified-HLJDD solutions under the positive and negative ion modes are shown in Figures 2(a)–2(d). To further investigate the degree of similarity and differences between the two groups, a loading plot of PLS-DA and S-plot of OPLS-DA were carried out. These plots are used to show differences in patterns of small samples in scatterplot positions close to each other, where farther distances indicate greater differences between samples (Figures 2(e) and 2(f)). The volcano plot showed all the significantly changed metabolites between HLJDD and modified-HLJDD solutions (Figures 2(g)), and we list Rt, *m*/*z*, and the proposed identity of the main significantly changed metabolites (VIP > 1 and *p* < 0.05) in Tables 1 and 2. We found that modified-HLJDD increased the concentrations of cyclic AMP, L-glutamine, adenine, L-pyroglutamic acid, adenosine, L-glutamate, and L-asparagine, as compared with HLJDD (Table 1). Besides, the metabolites, such as baicalein, wogonin, and baicalin, were decreased in the modified-HLJDD, as compared with HLJDD, consistent with the exclusion of Radix Scutellariae in modified-HLJDD (Table 2). To further explore the metabolic pathways involved in the significantly different metabolites between HLJDD and modified-HLJDD, the selected significant metabolites were mapped to known metabolic relation network (KEGG), PubChem, Match, and HMDB databases. The six top metabolic pathways constructed with the corresponding selected metabolites are shown as a bubble plot in Figure 2(h) and were as follows: flavone and flavonol biosynthesis; alanine, aspartate, and glutamatergic metabolism; arginine and proline metabolism; isoquinoline alkaloid biosynthesis; riboflavin metabolism; and tyrosine metabolism.

**Figure 3:**
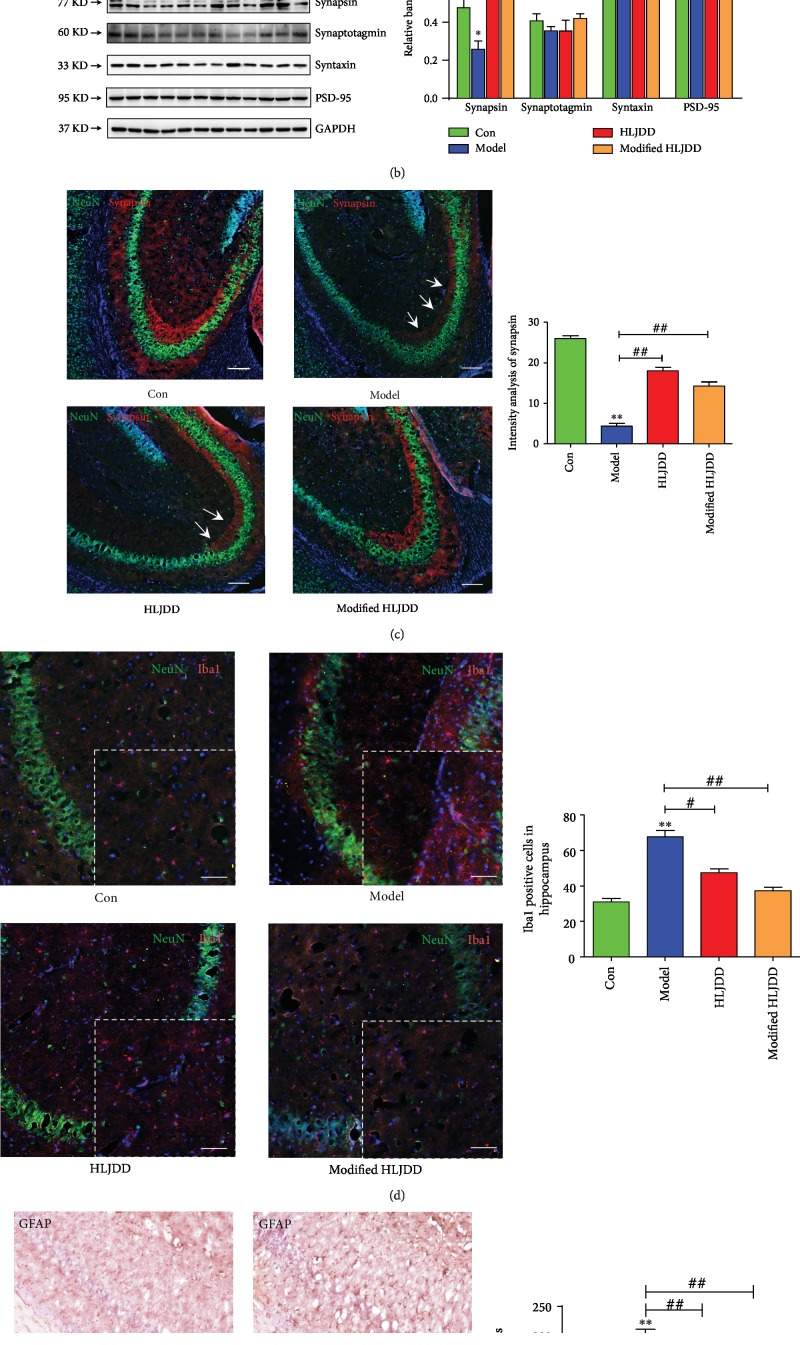
Modified-HLJDD ameliorates glutamatergic synaptic transmission in the hippocampus in an AD mouse model. (a) Effect of HLJDD and modified-HLJDD on glutamatergic receptor expression determined by Western blotting. (b) Effect of HLJDD and modified-HLJDD on synaptic protein expression determined by Western blotting. (c) Immunofluorescent staining of NeuN and synapsin in the hippocampus. Scale bar, 100 *μ*m. (d) Immunofluorescent staining of NeuN and Iba1 in the hippocampus. Scale bar, 100 *μ*m. (e) Immunohistochemical staining of GFAP in the hippocampal CA1. Scale bar, 50 *μ*m. *n* = 6 per group. Western blotting results are from three of the nine mice in each group and are expressed as the mean ± SEM of three experiments. ^∗∗^*p* < 0.01, ^∗^*p* < 0.05 vs. control group; ^##^*p* < 0.01, ^#^*p* < 0.05 vs. model group; and ^&^*p* < 0.05 vs. HLJDD group. Statistical significance was determined by one-way ANOVA and Bonferroni tests as *post hoc* comparisons.

Compared with HLJDD, modified-HLJDD increased glutamate and aspartate expression, and the inferred metabolic pathways also suggest that modified-HLJDD promoted glutamatergic and aspartate metabolism. To test the results from UPLC-Q-TOF/MS, we examined the expression of glutamatergic receptors and excitatory synaptic proteins in the hippocampus. Here, we found that the expression of NMDA receptors (NR1, NR2A, and NR2B), rather than that of AMPA receptors (GluA1 and GluA2), was decreased in the A*β*_1-42_ oligomer-treated AD model (Figure 3(a)), which is consistent with previous reports [34, 35]. Modified-HLJDD increased NR1, NR2A, and NR2B expressions, while HLJDD only increased NR1 expression in the hippocampus (Figure 3(a)). Moreover, the promotion of expression of NMDA receptors was much more obvious upon modified-HLJDD treatment compared to that of HLJDD treatment. We also examined synaptic protein expression and found that synapsin expression was decreased in the A*β*_1-42_ oligomer-treated AD model, while HLJDD and modified-HLJDD increased synapsin expression in the hippocampus in AD model (Figure 3(b)). Since synapsin is involved in presynaptic glutamatergic release, our results also suggested that modified-HLJDD may contribute to glutamatergic metabolism in our AD model. However, syntaxin, synaptotagmin, and PSD-95 expressions showed no obvious changes in these four groups (Figure 3(b)). To further confirm the effects of HLJDD and modified-HLJDD on synapsin expression, we performed immunofluorescence and we also found that HLJDD and modified-HLJDD increased synapsin expression in the hippocampus (Figure 3(c)). Usually, A*β*_1-42_ oligomer injection can induce reactive microgliosis and astrogliosis [36], and here, we also indicated that microgliosis and astrogliosis existed in our A*β*_1-42_ oligomer-treated AD model; importantly, HLJDD and modified-HLJDD suppressed reactive microgliosis and astrogliosis (Figures 3(d) and 3(e)). In addition, modified-HLJDD showed much more obvious effects on suppressing the astrogliosis, as compared with HLJDD (Figure 3(e)).

These results suggest that modified-HLJDD modulate glutamatergic metabolism and transmission in the A*β*_1-42_ oligomer-treated AD model.

### 3.3. Metabolomic Analysis of HLJDD and Modified-HLJDD Treatments on Hippocampal Metabolites in AD Model

**Figure 4:**
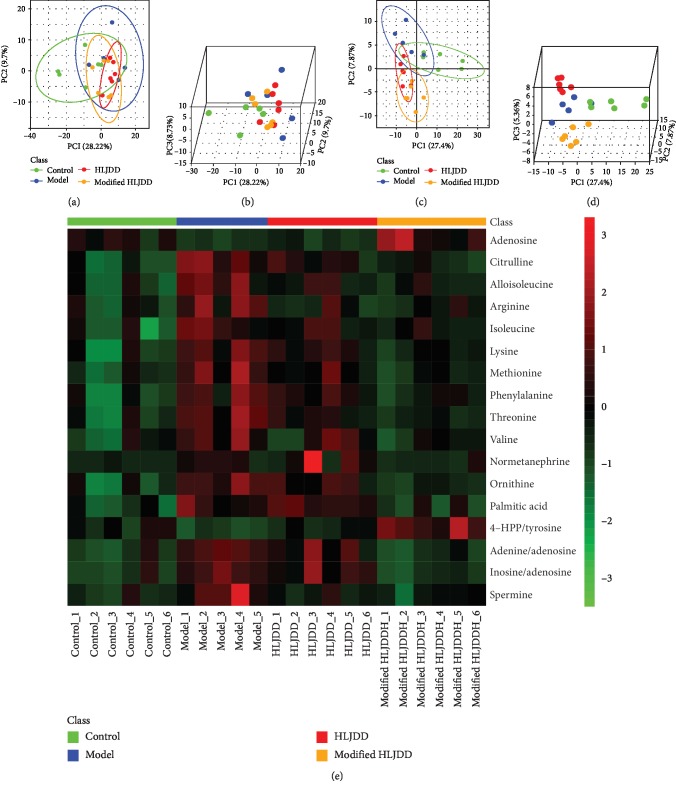
Metabolomic analysis of HLJDD and modified-HLJDD treatments on hippocampal metabolites in an AD model. (a, b) Overview of metabolic profiles of all the samples using PCA score plot. (c, d) PLS-DA score plot revealing classifications of the subjects. (e) The *Z*-score plot showing the relative variations of each individual metabolite across all the groups in the form of a heatmap.

Using UPLC-Q-TOF/MS, we have suggested partial significant metabolites between HLJDD and modified-HLJDD that may contribute to their different effects in our AD mouse model. To further explore an underlying mechanism, we examined the effects of HLJDD and modified-HLJDD on hippocampal metabolites in the A*β*_1-42_ oligomer-treated AD model. In this study, we used GC-TOF/MS for metabolomic analysis, and the annotated metabolites and their chemical classes are illustrated in Supplementary Fig. [Supplementary-material supplementary-material-1] and [Supplementary-material supplementary-material-1]. We also provided global metabolic profiles for the subjects from each subgroup, as revealed by scores plotted with an unsupervised multivariate statistical PCA model (Supplementary Fig. [Supplementary-material supplementary-material-1]). The scores plotted—among controls and AD mice, AD mice and HLJDD treatment in AD mice, and AD mice and modified-HLJDD treatment in AD mice—with an OPLS-DA model are presented in Supplementary Fig. [Supplementary-material supplementary-material-1]. A distinct separation of metabolites among these four groups was found in both PCA and three-dimensional (3-D) PLS-DA score plots, which indicates the significantly different metabolic profiles (Figures 4(a)–4(d)). The *Z*-score plot showed the relative variations of each individual metabolite across all the groups in the form of a heatmap (Figure 4(e)).

**Figure 5:**
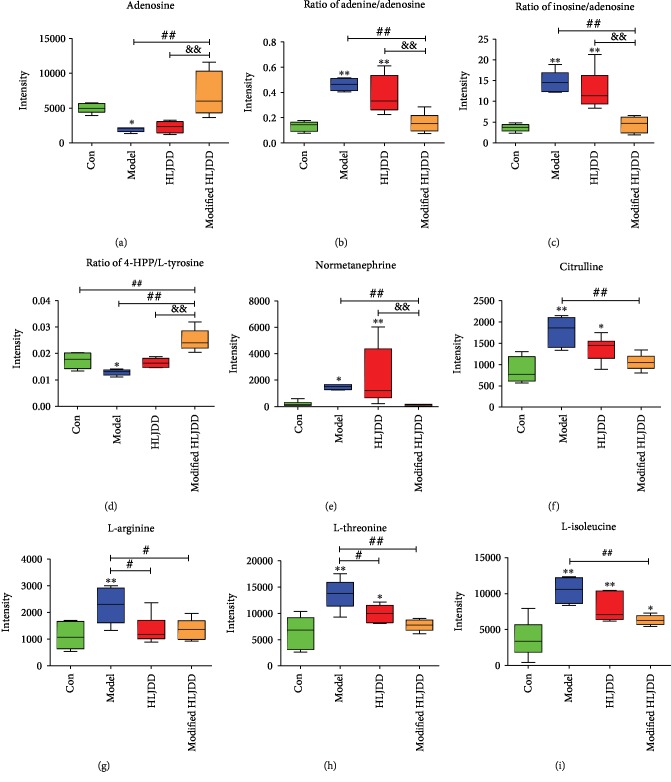
Differential metabolites upon HLJDD and modified-HLJDD treatments in an AD model. (a–i) Top-ranked differential metabolites among the four groups are shown. *n* = 6 for control, HLJDD, and modified-HLJDD groups. *n* = 5 for the AD model group. Results are expressed as the mean ± SEM. ^∗∗^*p* < 0.01, ^∗^*p* < 0.05 vs. control group; ^##^*p* < 0.01, ^#^*p* < 0.05 vs. model group; and ^&&^*p* < 0.01 vs. HLJDD group. Statistical significance was determined by one-way ANOVA and Bonferroni tests as *post hoc* comparisons.

In Figures 5(a)–5(i), we provide the top-nine-ranked differential metabolites among these four groups in the hippocampus. Because the relationship of metabolite connectivity can be used for depicting metabolite enzymatic activity, we used XploreMET 3.0 to provide metabolite ratios. The ratios of the two adjacent metabolites from the KEGG database were calculated. We found that hippocampal concentrations of adenosine and the ratio of 4-HPP/L-tyrosine were decreased in the A*β*_1-42_ oligomer-treated AD model, while modified-HLJDD treatment increased their concentrations in the AD model (Figures 5(a) and 5(d)). However, HLJDD treatment showed no obvious effects on these two metabolites in the AD model (Figures 5(a) and 5(d)). Correlated with the adenosine expression pattern, the ratio of adenine/adenosine and the ratio of inosine/adenosine were increased in the AD model, while modified-HLJDD treatment decreased these two ratios compared with the AD model and HLJDD treatment groups (Figures 5(b) and 5(c)), suggesting that adenosine was actually decreased in the hippocampus of the AD model. Previously, normetanephrine concentration was reported to be decreased in AD patients, and normetanephrine can promote microglia to uptake and degrade A*β* peptide [37]. However, we found that normetanephrine was increased in the hippocampus in our AD model, and modified-HLJDD treatment decreased these two ratios in the AD model (Figure 5(e)). We conclude that modified-HLJDD may attenuate the compensatory effect of normetanephrine in our AD model. In addition, we also found that amino acids—such as citrulline, L-arginine, L-threonine, and L-isoleucine—were increased in the hippocampus in our AD model, and HLJDD and modified-HLJDD treatments decreased these amino acid concentrations in our AD model (Figures 5(f)–5(i)).

Moreover, we also compared the significant metabolites between HLJDD and modified-HLJDD treatments in the hippocampus of our AD model. The scores plotted between HLJDD and modified-HLJDD treatments with an OPLS-DA model and permutation testing are shown in Supplementary Fig. [Supplementary-material supplementary-material-1] and [Supplementary-material supplementary-material-1]. Volcano plots and heatmaps were used to show the differential metabolites between these two groups (Supplementary Fig. [Supplementary-material supplementary-material-1] and [Supplementary-material supplementary-material-1]). As we have mentioned above, modified-HLJDD treatment increased adenosine and decreased the adenine/adenosine ratio and inosine/adenosine ratio compared with those in the HLJDD treatment in our AD model (Supplementary Fig. [Supplementary-material supplementary-material-1]-[Supplementary-material supplementary-material-1]). In addition, we also reported that modified-HLJDD increased the 4-HPP/L-tyrosine ratio and decreased palmitic acid, ornithine, the homogentisic acid/4-HPP ratio, and the ornithine/L-arginine ratio as compared with those in the HLJDD treatment (Supplementary Fig. [Supplementary-material supplementary-material-1]-[Supplementary-material supplementary-material-1]). These results suggest that modified-HLJDD treatment modulates some types of amino acid concentrations in the hippocampus in our AD model. Thus, the GC-TOF/MS results reveal a series of differential metabolites that may be responsible for the neuroprotective effects of modified-HLJDD in our AD model.

### 3.4. The Hippocampal ATPase/AMPK Cascade May Be Involved in the Modified-HLJDD-Mediated Changes in Adenosine Expression in AD Model

**Figure 6:**
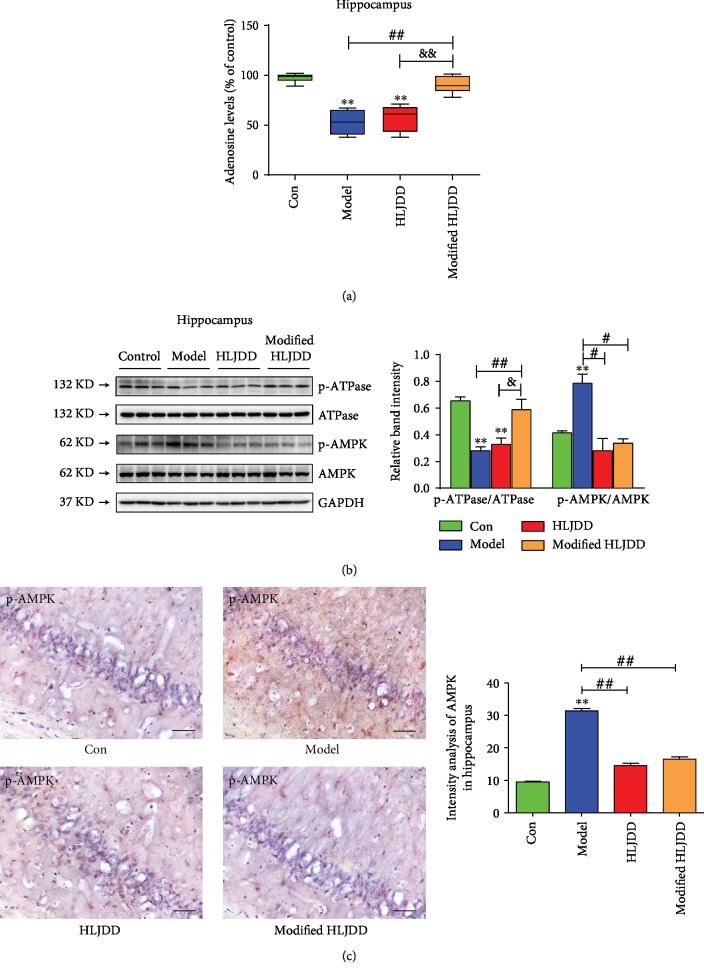
Effect of modified-HLJDD on ATPase and AMPK in the hippocampus in an AD model. (a) Effect of HLJDD and modified-HLJDD on the hippocampal adenosine level. (b) Effect of HLJDD and modified-HLJDD on ATPase and AMPK expression was determined by Western blotting. (c) Immunohistochemical staining of p-AMPK in the hippocampal CA1. Scale bar, 50 *μ*m. *n* = 6 per group. Western blotting results are from three of the nine mice in each group and are expressed as the mean ± SEM of three experiments. ^∗∗^*p* < 0.01 vs. control group; ^##^*p* < 0.01, ^#^*p* < 0.05 vs. model group; and ^&^*p* < 0.05 vs. HLJDD group. Statistical significance was determined by one-way ANOVA and Bonferroni tests as *post hoc* comparisons.

The GC-TOF/MS results suggest that the top-ranked differential metabolites, such as adenosine, may play an important role in the neuroprotective effects of modified-HLJDD in our AD model (Figures 5(a)–5(c)). To further confirm the GC-TOF/MS results and test our hypothesis on the role of adenosine in AD model, we firstly examined the adenosine level in the hippocampus, and we found that A*β*_1-42_ decreased the hippocampal adenosine level, and modified-HLJDD reversed the hippocampal adenosine level in A*β*_1-42_-treated mice (Figure 6(a)). This result was consistent with our GC-TOF/MS results (Figures 5(a)–5(c)). Next, we examined the hippocampal expression of ATPase and AMPK, mainly associated with ATP metabolism. ATPase is involved in ATP hydrolysis and energy biosynthesis, while AMPK is an energy sensor of metabolic stress mediated by the dysfunction of ATP biosynthesis, and both are important signaling molecules of ATP metabolism [38, 39]. Here, we found that ATPase phosphorylation was decreased, while AMPK phosphorylation was increased in the hippocampus in our AD model (Figure 6(b)), which is consistent with the decrease of ATPase with activation of AMPK reported in a previous study [40]. Modified-HLJDD increased both hippocampal adenosine and ATPase phosphorylation in A*β*_1-42_ only treated mice and A*β*_1-42_ plus HLJDD treated mice, suggesting that modified-HLJDD may increase ATP metabolism. We further confirmed our results by immunostaining, and we found that HLJDD and modified-HLJDD treatments suppressed AMPK activation in our AD model (Figure 6(c)). These results suggest that ATPase and AMPK may be involved in the modified-HLJDD promotion of adenosine revealed from our GC-TOF/MS results.

### 3.5. Effect of Modified-HLJDD on Gut Microbiota and Its Correlation with the Metabolomic Results in AD Model

We found that modified-HLJDD produced more adenosine in the hippocampus, revealed through metabolomic analysis, and we also inferred a related potential signaling pathway involved in this increased adenosine. Additionally, metabolomic changes in the brain may affect the gut microbiota via the brain-gut-microbiota axis, and gut microbiota contributes to the pathogenesis of AD [41]. Thus, we aimed to investigate whether A*β*_1-42_ oligomer injection impacts gut microbiota, whether modified-HLJDD treatment can induce changes of gut microbiota in the A*β*_1-42_ oligomer-treated AD model, and whether any changes in gut microbiota are correlated with significant hippocampal metabolites upon modified-HLJDD treatment in our AD model.

**Figure 7:**
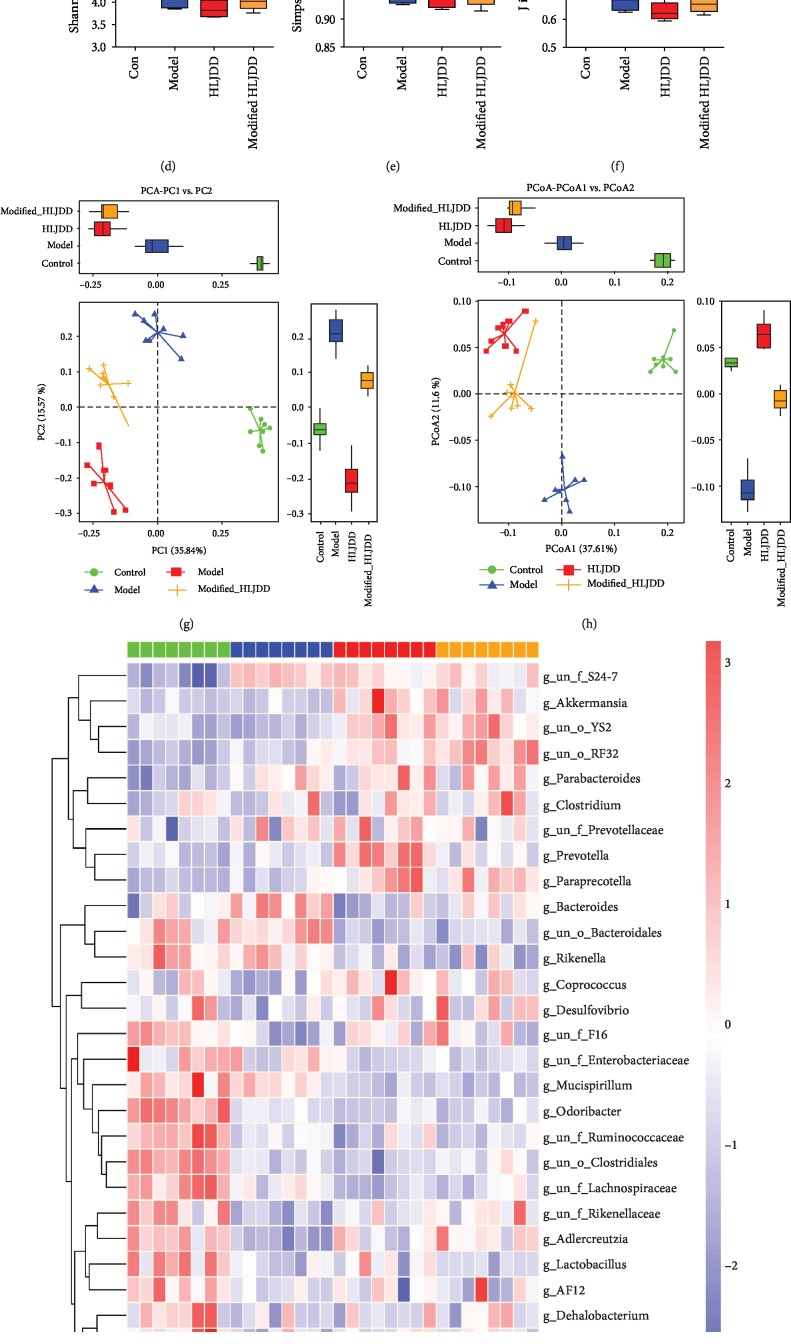
Effect of HLJDD and modified-HLJDD treatments on gut microbiota in an AD model. Six *α*-diversity of gut microbiota, including (a) observed species (OTUs), (b) Chao1, (c) ACE, (d) Shannon, (e) Simpson, and (f) J diversity indices, are shown. *β*-Diversity is shown in the fashion of (g) PCA plots and (h) PCoA plots. (i) The hierarchy cluster heatmap revealing the top 30 most abundant differentiated taxa at the genus level in feces samples. *n* = 8 per group. Results are expressed as the mean ± SEM. ^∗∗^*p* < 0.01, ^∗^*p* < 0.05 vs. control group. Statistical significance was determined by one-way ANOVA and Bonferroni tests as *post hoc* comparisons.

The *α*-diversity reflects the diversity of bacteria or species within a community or habitat and is mainly involved in the number of bacteria or species therein [42]. Six *α*-diversity measures were calculated, including observed species (OTUs), Chao1, ACE, Shannon, Simpson, and J diversity indices (Figures 7(a)–7(f)). We found that A*β*_1-42_ oligomer injection significantly decreased the *α*-diversity; however, both HLJDD and modified-HLJDD did not reverse this decrease. Regarding *β*-diversity, we found that PCA and principal coordinate analysis (PCoA) plots of Bray-Curtis dissimilarity among these groups showed that the dots of A*β*_1-42_ oligomer-treated mice were not close to those of controls, and both HLJDD and modified-HLJDD treatments were also separated from the AD model (Figures 7(g) and 7(h)). We also showed the analysis of similarities (ANOSIM), nonmetric multidimensional scaling (NMDS) analysis, and sample-species composition analysis among these four groups (Supplementary Fig. [Supplementary-material supplementary-material-1]-[Supplementary-material supplementary-material-1]). Thus, it is likely that A*β*_1-42_ oligomer injection induces a different gut microbiota composition compared with that of control mice. In addition, HLJDD and modified-HLJDD treatments may change the gut microbiota composition in our AD model.

**Figure 8:**
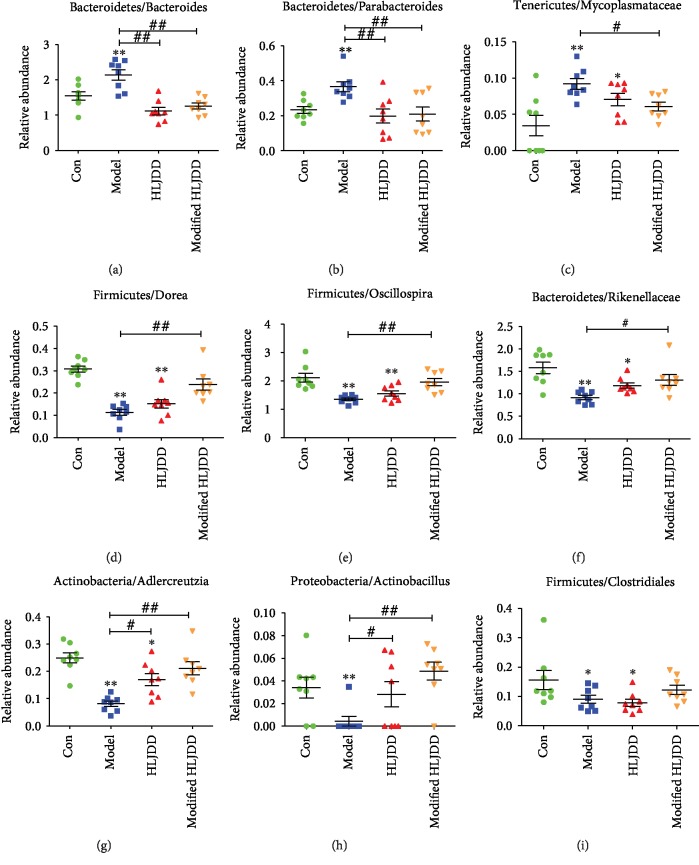
Differential microbiota upon HLJDD and modified-HLJDD treatments in an AD model. (a–i) Top-ranked differential microbiota among the four groups is shown. *n* = 8 per group. Results are expressed as the mean ± SEM. ^∗∗^*p* < 0.01, ^∗^*p* < 0.05 vs. control group; ^##^*p* < 0.01, ^#^*p* < 0.05 vs. model group. Statistical significance was determined by one-way ANOVA and Bonferroni tests as *post hoc* comparisons.

Next, we used 16S rDNA gene-sequencing to determine the alterations in the gut microbiota composition among these groups. The taxon abundance of each sample was classified in terms of phylum, class, order, family, genus, and species levels, mainly using the QIIME reference database. Concerning the genus abundance in feces samples, the hierarchy cluster heatmap revealed the top 30 most abundant differentiated taxa (Figure 7(i)). To be more specific, we further calculated multiple comparisons to show the conspicuous group differences at the phylum and genus levels. *Firmicutes* and *Bacteroidetes* were the two most dominant in these groups (Figure 8 and Supplementary Fig. [Supplementary-material supplementary-material-1]). The significantly increased genera in the A*β*_1-42_ oligomer-treated mice included *Bacteroides*, *Parabacteroides*, and *Mycoplasmataceae*, while the significantly decreased genera included *Dorea*, *Oscillospira*, *Rikenellaceae*, *Adlercreutzia*, *Actinobacillus*, *Clostridiales*, *Anaeroplasma*, *Lactobacillus*, *Odoribacter*, *Lachnospiraceae*, *Ruminococcaceae*, and *Enterobacteriaceae*. HLJDD and modified-HLJDD treatments significantly decreased the abundance of *Bacteroides* and *Parabacteroides*, while modified-HLJDD treatment also decreased the abundance of *Mycoplasmataceae* in the AD model (Figures 8(a)–8(c)). Regarding the decreased genera, modified-HLJDD treatment significantly increased the abundance of *Dorea*, *Oscillospira*, and *Rikenellaceae* in the AD model (Figures 8(d)–8(f)). Both HLJDD and modified-HLJDD treatments increased the *Adlercreutzia* and *Actinobacillus* abundance in the AD model (Figures 8(g) and 8(h)). However, both HLJDD and modified-HLJDD treatments showed no obvious effects on the abundance of *Clostridiales*, *Lactobacillus*, *Odoribacter*, *Lachnospiraceae*, *Ruminococcaceae*, and *Enterobacteriaceae*, except only that HLJDD treatment increased the abundance of *Anaeroplasma* in the AD model (Figure 8(i) and Supplementary Fig. [Supplementary-material supplementary-material-1]-[Supplementary-material supplementary-material-1]).

**Figure 9:**
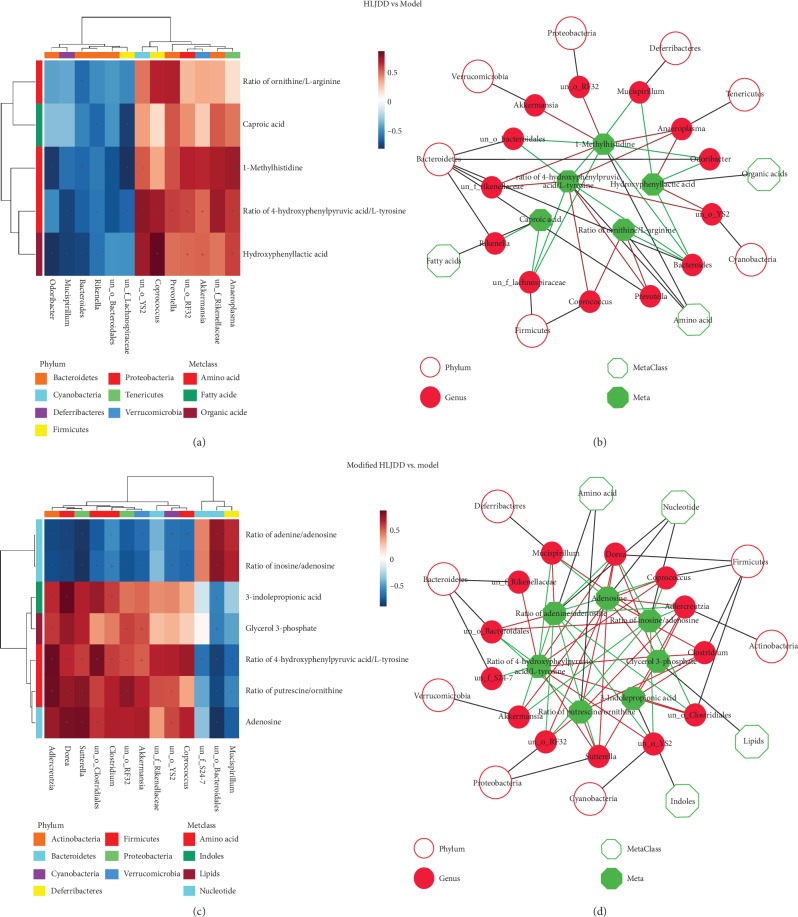
The correlation between significant hippocampal metabolites and gut microbiota in the AD model. (a, b) The correlation between significant hippocampal metabolites and gut microbiota is shown for HLJDD vs. AD model in the form of a heatmap and network diagram. (c, d) The correlation between significant hippocampal metabolites and gut microbiota is shown for modified-HLJDD vs. AD model in the form of a heatmap and network diagram.

Using GC-TOF/MS, we have indicated the differential hippocampal metabolites upon modified-HLJDD treatment in our AD model. To further reveal the correlation between hippocampal metabolomic results and gut microbiota upon modified-HLJDD treatment, we performed the Spearman correlation analysis of significant hippocampal metabolites and gut microbiota between controls and AD mice, AD mice and HLJDD treatment in AD mice, AD mice and modified-HLJDD treatment in AD mice, and HLJDD and modified-HLJDD treatments in AD mice. The correlation between significant hippocampal metabolites and gut microbiota is shown in the form of a heatmap, network diagram, and chord diagram (Figures 9(a) and 9(b) for HLJDD vs. AD model, Figures 9(c) and 9(d) for modified-HLJDD vs. AD model, Supplementary Fig. [Supplementary-material supplementary-material-1]-[Supplementary-material supplementary-material-1] for AD model vs. control, and Supplementary Fig. [Supplementary-material supplementary-material-1]-[Supplementary-material supplementary-material-1] for modified-HLJDD vs. HLJDD). Since adenosine-related signaling is the most important pathway screened by our metabolomic analysis, we focused on the related gut microbiotas involved in the adenosine pathway. Between the AD model and control, we found that the abundance of *Arthromitus* (*R* = 0.74, *p* = 0.009), *Dorea* (*R* = 0.679, *p* = 0.022), *Rikenellaceae* (*R* = 0.645, *p* = 0.032), and *Oscillospira* (*R* = 0.618, *p* = 0.043) at the level of genus was closely correlated with adenosine. Additionally, between modified-HLJDD in AD model and the AD model, the abundance of *Sutterella* (*R* = 0.836, *p* = 0.001), *Dorea* (*R* = 0.825, *p* = 0.002), *Adlercreutzia* (*R* = 0.752, *p* = 0.008), *Mucispirillum* (*R* = −0.705, *p* = 0.015), *Akkermansia* (*R* = 0.709, *p* = 0.015), *Clostridium* (*R* = 0.7, *p* = 0.016), *RF32* (*R* = 0.691, *p* = 0.019), *Coprococcus* (*R* = 0.682, *p* = 0.021), and *Clostridiales* (*R* = 0.636, *p* = 0.035) at the level of genus was closely correlated with adenosine. HLJDD treatment showed no significant correlation between gut microbiota and adenosine, which is consistent with our metabolomic results that HLJDD treatment showed no significant effect on hippocampal adenosine concentration in our AD model. Regarding the ratios of adenine/adenosine and inosine/adenosine, we also revealed that *Dorea* is the most closely related microbiota with adenosine upon modified-HLJDD treatment in the A*β*_1-42_ oligomer-induced AD mice model.

## 4. Discussion

A*β* is the hallmark of AD pathology; however, recently, several immunotherapeutic strategies targeting formed A*β* plaque in the brain have proven to be failed in the clinical trials [18, 19]. These evidences suggest us that therapeutic strategies targeting A*β* may need to be put forward. HLJDD is a classic formula of TCM that is commonly used to treat neurologic diseases, such as dementia and ischemic stroke, in the Far East. The active compounds of HLJDD include berberine, baicalin, baicalein, and geniposide. Previously, another modified formulation of HLJDD (composed of Rhizoma Coptidis, Cortex Phellodendri, and Fructus Gardeniae without Radix Scutellariae) has been reported to ameliorate the learning and memory impairment in 3xTg AD mice and lessen A*β* plaque burden *in vitro* and *in vivo* [24, 43]. In the present study, according to our clinical practice and the study of Durairajan et al. [43], we generated a novel modified-HLJDD (also named Jie-Du-Hua-Yu Decoction) (HLJDD without Radix Scutellariae but with added Salvia miltiorrhiza, Curcuma longa L., and Acorus tatarinowii). We evaluated the effects of modified-HLJDD on the learning and memory impairment in our AD model, and we also explored its mechanism of action via application of metabolomic and gut microbiota analysis.

Nowadays, there are multiple types of AD animal models, including the A*β*_1-42_ oligomer-induced model, D-galactose and aluminum chloride-induced model, and transgenic models, such as APP/PS1, 3xTg AD, and 5xFAD. As we know, the dysfunction of synaptic plasticity appears before A*β* plaque formation and contributes significantly to AD pathogenesis. Intracerebroventricular microinjection of A*β* can induce cognitive dysfunction, and it is also a common AD animal model to examine the effect of A*β* on synaptic plasticity [44–46]. In the present study, we aimed to examine the effects of HLJDD and its modification on synaptic plasticity, so we chose the A*β*_1-42_ oligomer-induced AD model for this purpose. We indicated that microinjection of A*β*_1-42_ oligomer induced memory deficiency in mice. Moreover, A*β*_1-42_ oligomer decreased NMDA receptors and synapsin expression, suggesting that A*β*_1-42_ oligomer actually induced dysfunctional excitatory synaptic plasticity in mice. Thus, this model is suitable for studying potential effects of modified-HLJDD.

Subsequently, we confirm the main compositions after modification of HLJDD using UPLC-Q-TOF/MS. We found that modified-HLJDD mainly affect the glutamatergic and aspartate metabolism, as L-glutamine, L-pyroglutamic acid, L-glutamate, and L-asparagine were increased compared with HLJDD. Another metabolic pathway is flavone and flavonol biosynthesis. Since we exclude Radix Scutellariae in the modified-HLJDD, we found that concentrations of baicalein, wogonin, and baicalin—which are the main ingredients of Radix Scutellariae—were significantly decreased. These results suggest that Radix Scutellariae in HLJDD contributes significantly to the flavone metabolism. Referring to comparison of the neuroprotective effects of modified-HLJDD with HLJDD, we found that modified-HLJDD showed much more obvious effects on ameliorating memory deficiency in MWM and T-/Y-maze tests in A*β*_1-42_ oligomer-induced AD model (Figure 1). In addition, we also found that modified-HLJDD was much more effective in promoting NMDA receptor expression, suppressing astrogliosis, and increasing the adenosine level in the hippocampus. Considering that another modification of HLJDD shows benefits in 3xTg AD mice, our modified-HLJDD may exert much more neuroprotective effects in A*β*_1-42_ oligomer-induced AD model. In the modification of HLJDD, we excluded Radix Scutellariae, regarded as meaningless even harmful in treating AD [24], and we added Salvia miltiorrhiza, Curcuma longa L., and Acorus tatarinowii. The active ingredients in these three herbs (such as tanshinone IIA, curcumin, *β*-asarone) have been widely proved to exert therapeutic effects in various AD models [47–51]. Thus, we could conclude that our modification of HLJDD was much more effective than HLJDD in A*β*_1-42_ oligomer-induced AD model. Since HLJDD improved mice performance in the open field test (Supplementary Fig. [Supplementary-material supplementary-material-1]), and active ingredients in Radix Scutellariae mainly exert anxiolytic effects [52], suggesting that HLJDD may improve A*β*_1-42_ oligomer-induced anxiety-related behavior, as stated in the previous literature [53].

In this study, we draw out two possible mechanisms underlying the modified-HLJDD-ameliorating memory deficits in our AD model using metabolomic methods. On the one hand, our UPLC-Q-TOF/MS results suggest that modified-HLJDD may affect the glutamatergic and aspartate metabolism, as compared with HLJDD (Table 1 and Figure 2(h)). We indicated that modified-HLJDD reversed A*β*_1-42_ oligomer injections and decreased NMDA receptors (especially NMDAR1, NMDAR2A, and NMDAR2B) and synapsin expression in the hippocampus, suggesting that modified-HLJDD improves A*β*_1-42_ oligomer-induced dysfunction of glutamatergic synaptic transmission. We also found that modified-HLJDD treatment promoting NMDA receptors and synapsin expressions was significantly greater than HLJDD treatment (Figures 3(a) and 3(b)), suggesting that modified-HLJDD may be much more effective in modulating glutamatergic transmission in A*β*_1-42_ oligomer-induced AD model. These effects may partially account for modified-HLJDD effects on the behavioral deficits in AD model.

On the other hand, we also examined which metabolites in the hippocampus were responsible for the dysfunctional synaptic plasticity using GC-TOF/MS. We discovered many differential metabolites among these groups and then focused on the adenosine-related pathway since it was among the metabolites that were most changed. As we mentioned above, adenosine is a ubiquitous neuromodulator in the central nervous system. Adenosine can be generated from adenine nucleosides via 5′-nucleotidase enzymes intracellularly or extracellularly [8], and it regulates neuronal survival, glutamate and aspartate release, and acetylcholine release [9–11]. Previously, adenosine has been proposed to be neuroprotective against AD [12], and its function mainly involves the binding of adenosine receptors. Adenosine is an important source of purine, and purinergic signaling is also an important therapeutic target in AD, which mainly involves the effects of ATP binding to purinergic receptors [54]. This family of receptors has been shown to regulate neuroinflammation, synaptic plasticity, learning, and memory [55, 56]. In the central nervous system, ATP has a proinflammatory and proapoptotic action, and adenosine is involved in neuroprotection via several mechanisms [57]. ATP and ADP are hydrolyzed by ATPase and 5′-nucleotidase into adenosine, and this conversion is disrupted in AD [58]. Usually, oxidative stress decreases ATP production by impairing mitochondria, and mitochondrial dysfunction decreases energy metabolism in AD animal models, suggesting that adenosine is also decreased in AD [58]. In our study, we found that modified-HLJDD increased both hippocampal adenosine and ATPase phosphorylation in A*β*_1-42_ only treated mice and A*β*_1-42_ plus HLJDD treated mice. These results suggest that modified-HLJDD may improve dysfunctional ATP metabolism and ameliorate the oxidative stress and mitochondrial dysfunction in AD model, which may be partially explained by modified-HLJDD suppressing reactive microgliosis and astrogliosis in the hippocampus of AD model. We conclude that these two mechanisms may be linked, since NMDA receptors can decrease adenosine kinase activity and evoke adenosine accumulation [59, 60], and adenosine can also regulate glutamatergic transmission [61]. According to this previous study and the results of the present study, NMDA receptors were decreased in the hippocampus in AD model, and we also concluded that the downstream adenosine pathway may also be decreased. The decreased ATPase activity also supports our hypothesis that NMDA receptors and adenosine signaling are downregulated in the hippocampus in the A*β*_1-42_ oligomer-induced AD model. Additionally, modified-HLJDD promotes NMDA receptors and ATP metabolism while attenuating cognitive impairment in our AD model. However, further study needs to clarify the relation between NMDA receptors and adenosine signaling upon modified-HLJDD treatment in AD model.

AMPK is an energy sensor and regulator of dysfunctional ATP biosynthesis-mediated metabolic stress. As we know, the gamma subunit of AMPK holds adenine-nucleotide-binding sites, which allow the sensing of intracellular levels of AMP, ADP, and ATP [62]. Thus, modification of the intracellular AMP/ATP ratio mediated by any energetic stress will enhance the activation of AMPK [62]. Moreover, AMPK is crucial to maintaining intracellular neuronal energy metabolism upon synaptic activation through adapting the glycolysis/mitochondrial respiration rate, and neuronal AMPK has been reported to be overly activated in the brain of AD patients [63, 64]. In the present study, we also found that A*β*_1-42_ oligomer injection induces overactivation of AMPK in the hippocampus, suggesting that A*β*_1-42_ oligomers lead to disrupted energy metabolism. According to our metabolic results, we conclude that the dysfunction of energy metabolism may be related to ATP exhaustion and decreased adenosine in the hippocampus. Both HLJDD and modified-HLJDD reversed AMPK overactivation, suggesting that these two treatments may ameliorate the disruption in energy metabolism in our AD model. However, considering that HLJDD did not increase adenosine concentration in the hippocampus, we conclude that there may be other pathways regulating HLJDD-decreased AMPK activity.

A*β*_1-42_ oligomer-induced metabolomic changes in the brain may affect the gut microbiota via the brain-gut-microbiota axis, and gut-brain signaling is required for sustaining energy homeostasis [65]. Importantly, dysfunction of gut microbiota contributes to the pathogenesis of AD [41]. Several bacterium taxa have been reported to be correlated with AD, such as *Citrobacter rodentium*, *Chlamydia pneumoniae*, and *Helicobacter* [66–68]. Concerning the altered gut microbiota in the A*β*_1-42_ oligomer-induced AD model, we also found that a series of bacterium taxa were altered. In our study, *Firmicutes*, *Bacteroidetes*, *Proteobacteria*, and *Actinobacteria* were the dominant bacteria at the phylum level, which is consistent with the altered gut microbiota in AD patients [69, 70]. At the genus level, we identified that *Bacteroides*, *Parabacteroides*, and *Mycoplasmataceae* were significantly increased in the AD model, while both HLJDD and modified-HLJDD decreased their concentrations. The abundance of *Bacteroides* was increased in the gut microbiome of AD patients [70]. Recently, as a *Bacteroidetes* member, gram-negative facultative anaerobe *Bacteroides fragilis* has been reported to play a pathogenic role in AD through secreting proinflammatory lipopolysaccharides and consequently inducing NF-*κ*B-miRNA-directed gene expression [71]. *Parabacteroides* is an anti-inflammatory type of bacteria that produces short-chain fatty acids (SCFAs), and current studies have not revealed the connection between *Parabacteroides* and cognitive impairment in humans. However, HLJDD has been previously shown to increase the abundance of gut *Parabacteroides* in a type-2 diabetes mellitus model [28]. We conclude from these results that HLJDD and modified-HLJDD may exert anti-inflammatory effects by downregulating these kinds of gut bacteria in the A*β*_1-42_ oligomer-induced AD model. We also revealed other downregulated gut microbiota in our AD model—such as *Dorea*, *Oscillospira*, and *Adlercreutzia*—which mainly belong to the *Firmicutes* phylum. Among these gut microbiota compositions, at the genus level, *Rikenellaceae*, *Lactobacillus*, *Lachnospiraceae*, and *Ruminococcaceae* have been reported to be decreased in AD animal models [72–75], while *Odoribacter* has been reported to be increased in AD models [76]. In our present study, only modified-HLJDD increased the abundance of *Rikenellaceae*, suggesting its neuroprotective effects in our AD model. *Oscillospira* can degrade host glycans and is positively associated with leanness or lower body mass index in humans [77]. Its abundance is decreased in inflammatory diseases, and, in the present study, we found that modified-HLJDD treatment increased *Oscillospira* abundance in our AD model. Since there was no evidence to support that *Adlercreutzia* and *Actinobacillus* are correlated with cognitive impairment or aging, future studies will be needed to further investigate these two gut bacteria in AD models.

In the present study, we first correlated the hippocampal metabolites with gut microbiota in our AD model, and we screened—at the genus level—that the abundance of *Dorea* was positively correlated with adenosine. Although *Dorea* has not been reported to participate in AD, it showed less abundance in the gut microbiota of heart failure patients [78]. In addition, *Dorea* has been shown to be associated with leanness and levodopa metabolism in Parkinson's disease [79, 80]. It is noteworthy that *Dorea* may be involved in the energy metabolism underlying leanness, suggesting that it may participate in ATP metabolism. Further studies are needed to elucidate the regulation of gut microbiota *Dorea* on ATP-related metabolism in AD models.

Previously, according to the traditional Chinese medicine theories and our clinical practice, we identified that the formula of HLJDD is an efficacious prescription in treating patients with AD. In 2014, we noticed the study of Durairajan et al. about HLJDD. In their study, Durairajan et al. examined the effects of each herb of the HLJDD in AD model [24]. Using the N2a-SwedAPP cellular model, they found out that the extracts from Rhizoma Coptidis and Cortex Phellodendri could reduce the generation of A*β* peptide; the extracts from Fructus Gardeniae showed no obvious change on the A*β* peptide production. However, Radix Scutellariae and its active ingredients, baicalein, significantly increased APP modulation and A*β* peptide production, suggesting that Radix Scutellariae may be detrimental to the AD pathology. To improve the efficacy of HLJDD, we excluded Radix Scutellariae from the HLJDD. According to our clinical practice, we further added Salvia miltiorrhiza, Curcuma longa L., and Acorus tatarinowii, which are beneficial to A*β* peptide clearance in the animal model [20, 81–83]. Thus, we generated our newly modified formula of HLJDD, which is HLJDD without Radix Scutellariae, and with the addition of Salvia miltiorrhiza, Curcuma longa L., and Acorus tatarinowii. In this study, we indicated that the effects of modified-HLJDD were much more obvious as compared with HLJDD in A*β*-induced AD mouse model, and we also identified the underlying metabolic and microbial mechanisms of modified-HLJDD in AD mouse model. We have to say that our study at least confirms the effects of modified-HLJDD reported by Durairajan et al. (HLJDD without Radix Scutellariae); however, it is hard to say whether Salvia miltiorrhiza, Curcuma longa L., and Acorus tatarinowii synergize with the other herbs. Hence, further studies are needed to screen each of the herbs in the modified-HLJDD separately and determine what effects they have with regard to control and at the time of treatment and finally to identify what the effective components are in the modified-HLJDD.

## 5. Conclusion

In this study, we found that modified-HLJDD attenuates cognitive impairment in the A*β*_1-42_ oligomer-induced AD mouse model. Using metabolomic and gut microbiota analyses, we revealed that the neuroprotective mechanism of modified-HLJDD may involve modulation of NMDA receptor-mediated glutamatergic transmission and adenosine-related signaling pathway. We also identify significant gut microbiota that may be involved in the adenosine pathway. Thus, modified-HLJDD is a potential therapeutic drug for AD.

## Figures and Tables

**Table 1:** The proposed identity of main significantly increased metabolites in modified-HLJDD solution as compared with HLJDD solution (VIP > 1 and *p* < 0.05).

No.	Rt (sec)	*m*/*z*	Scan mode	Proposed identity	Fold change
1	283.866	265.04975	ESI-	Uridine	8.98^∗∗^
2	335.147	145.01406	ESI-	Alpha-ketoglutarate	8.40^∗∗^
3	401.3015	274.10344	ESI-	Ribothymidine	7.26^∗∗^
4	447.433	229.03427	ESI-	Gallic acid	7.11^∗∗^
5	252.0905	736.18676	ESI+	Salvianolic acid L	5.86^∗∗^
6	429.9505	739.13038	ESI-	Salvianolic acid L	2.21^∗∗^
7	329.3	330.05854	ESI+	Cyclic AMP	5.09^∗∗^
8	240.281	328.04339	ESI-	Cyclic AMP	2.33^∗∗^
9	332.147	274.09177	ESI+	5′-Deoxyadenosine	4.26^∗∗^
10	361.654	250.09206	ESI-	5′-Deoxyadenosine	2.68^∗∗^
11	349.5	145.0617	ESI-	L-glutamine	4.89^∗∗^
12	349.445	147.07597	ESI+	L-glutamine	4.16^∗∗^
13	179.804	359.07497	ESI-	Rosmarinic acid	4.49^∗∗^
14	145.342	134.04704	ESI-	Adenine	3.59^∗∗^
15	144.513	136.06145	ESI+	Adenine	2.43^∗∗^
16	170.723	130.08876	ESI-	L-isoleucine	3.58^∗∗^
17	271.469	128.03637	ESI-	L-pyroglutamic acid	2.78^∗∗^
18	369.372	259.09214	ESI+	L-pyroglutamic acid	2.65^∗∗^
19	147.5605	279.03784	ESI-	Thymidine	2.41^∗∗^
20	370.018	250.092	ESI+	N-Acetyl-L-glutamate	2.21^∗∗^
21	355.314	188.05576	ESI-	N-Acetyl-L-glutamate	1.91^∗∗^
22	218.562	242.07682	ESI-	Cytidine	2.17^∗∗^
23	218.9115	244.0919	ESI+	Cytidine	1.64^∗∗^
24	369.475	268.10212	ESI+	Adenosine	2.12^∗∗^
25	330.727	236.07927	ESI+	N-Acetyl-L-aspartic acid	2.05^∗∗^
26	360.825	174.04019	ESI-	N-Acetyl-L-aspartic acid	1.39^∗∗^
27	316.1155	289.07948	ESI+	5-Methylcytosine	2.03^∗∗^
28	190.296	439.09881	ESI-	Gardenoside	2.00^∗∗^
29	293.1405	306.04803	ESI+	Cytidine 2′,3′-cyclic phosphate	1.94^∗∗^
30	250.9335	104.10697	ESI+	Choline	1.37^∗∗^
31	368.308	146.04593	ESI-	L-glutamate	1.53^∗∗^
32	368.0045	148.05971	ESI+	L-glutamate	1.35^∗∗^
33	251.085	130.08726	ESI-	L-leucine	1.50^∗^
34	288.0925	158.09171	ESI+	L-citrulline	1.49^∗^
35	101.406	168.06609	ESI-	Pyridoxine	1.39^∗^
36	352.4745	131.04657	ESI-	L-asparagine	1.33^∗∗^
37	352.261	133.06052	ESI+	L-asparagine	1.28^∗∗^
38	407.805	362.04978	ESI-	Guanosine 5′-monophosphate (GMP)	1.19^∗^
39	232.449	164.07138	ESI-	L-phenylalanine	1.19^∗^

^∗^ *p* < 0.05 and ^∗∗^*p* < 0.01.

**Table 2:** The proposed identity of main significantly decreased metabolites in modified-HLJDD solution as compared with HLJDD solution (VIP > 1 and *p* < 0.05).

No.	Rt (sec)	*m*/*z*	Scan mode	Proposed identity	Fold change
1	41.926	255.2318365	ESI-	Palmitic acid	0.025^∗∗^
2	104.5675	303.0493473	ESI-	Taxifolin	0.027^∗∗^
3	40.3525	331.0809904	ESI+	Baicalein	0.035^∗∗^
4	204.4315	549.1595046	ESI+	Puerarin xyloside	0.036^∗∗^
5	204.029	547.1436023	ESI-	Puerarin xyloside	0.055^∗∗^
6	44.7035	301.034413	ESI-	Quercetin	0.04^∗∗^
7	41.5445	301.0704952	ESI+	Diosmetin	0.04^∗∗^
8	93.245	351.017593	ESI-	Glycitein	0.04^∗∗^
9	179.129	283.0598699	ESI-	Glycitein	0.07^∗∗^
10	35.1845	345.0968707	ESI+	Prunetin	0.047^∗∗^
11	40.656	283.2638094	ESI-	Wogonin	0.055^∗∗^
12	220.9455	447.0925415	ESI+	Baicalin	0.06^∗∗^
13	78.325	269.0918269	ESI+	Inosine	0.06^∗∗^
14	213.5715	355.0439436	ESI-	Phenolphthalein	0.089^∗∗^
15	44.9385	287.0909411	ESI+	4′,5-Dihydroxy-7-methoxyflavanone	0.097^∗∗^
16	145.5845	417.1185387	ESI+	Daidzin	0.10^∗∗^
17	245.733	463.0857816	ESI-	Isoquercitin	0.13^∗∗^
18	177.391	253.0495934	ESI-	Chrysin	0.16^∗∗^
19	113.6665	433.1474268	ESI+	Indolelactic acid	0.18^∗∗^
20	42.905	271.0599859	ESI+	Apigenin	0.19^∗∗^
21	91.8115	433.1133567	ESI+	Apigenin 7-glucoside	0.20^∗^
22	41.536	359.1128039	ESI+	Berberine	0.24^∗∗^
23	36.886	286.0792349	ESI+	Flavone	0.28^∗∗^
24	102.493	215.0338159	ESI-	3-Chloro-L-tyrosine	0.33^∗∗^
25	406.4715	323.0268477	ESI-	Uridine 5′-monophosphate (UMP)	0.55^∗∗^
26	26.0535	167.037797	ESI-	Homogentisic acid	0.70^∗∗^
27	396.7205	348.0703451	ESI+	Adenosine monophosphate (AMP)	0.63^∗∗^
28	395.9115	387.0914333	ESI-	Adenosine monophosphate (AMP)	0.77^∗∗^

^∗^ *p* < 0.05 and ^∗∗^*p* < 0.01.

## Data Availability

The data used to support the findings of this study are available from the corresponding author upon request.

## Abbreviations

AMPK: AMP-activated protein kinase
GC-Q-TOF/MS: Gas chromatography quadrupole time of flight mass spectrometry
HLJDD: Huang-Lian-Jie-Du-Decoction
OPLS- DA: Orthogonal partial least-square discriminant analysis
OTUs: Operational taxonomic units
PCA: Principal component analysis
PCoA: Principal coordinate analysis
PLS-DA: Partial least-square discriminant analysis
TCI: Total ion chromatograms
UPLC-Q-TOF/MS: Ultraperformance liquid chromatography quadrupole time of flight mass spectrometry
VIP: Variable importance in the projection.

## Supplementary Materials

Supplementary Materials Supplementary Materials mainly contain the additional behavioral tests, the additional metabolic profiles, and metabolic and microbial differences between modified-HLJDD and HLJDD.

## References

[1] Fagan A. M., Mintun M. A., Mach R. H. (2006). Inverse relation between in vivo amyloid imaging load and cerebrospinal fluid A*β*_42_ in humans. *Annals of Neurology*.

[2] Tu S., Okamoto S., Lipton S. A., Xu H. (2014). Oligomeric A*β*-induced synaptic dysfunction in Alzheimer’s disease. *Molecular Neurodegeneration*.

[3] Shankar G. M., Walsh D. M. (2009). Alzheimer’s disease: synaptic dysfunction and A*β*. *Molecular Neurodegeneration*.

[4] Arbel-Ornath M., Hudry E., Boivin J. R. (2017). Soluble oligomeric amyloid-*β* induces calcium dyshomeostasis that precedes synapse loss in the living mouse brain. *Molecular Neurodegeneration*.

[5] Reinders N. R., Pao Y., Renner M. C. (2016). Amyloid-*β* effects on synapses and memory require AMPA receptor subunit GluA3. *Proceedings of the National Academy of Sciences of the United States of America*.

[6] Cai Q., Tammineni P. (2017). Mitochondrial aspects of synaptic dysfunction in Alzheimer’s disease. *Journal of Alzheimer's Disease*.

[7] Forny-Germano L., Lyra e silva N. M., Batista A. F. (2014). Alzheimer’s disease-like pathology induced by amyloid-*β* oligomers in nonhuman primates. *Journal of Neuroscience*.

[8] Zimmermann H. (2000). Extracellular metabolism of ATP and other nucleotides. *Naunyn-Schmiedeberg's Archives of Pharmacology*.

[9] Brand A., Vissiennon Z., Eschke D., Nieber K. (2001). Adenosine A_1_ and A_3_ receptors mediate inhibition of synaptic transmission in rat cortical neurons. *Neuropharmacology*.

[10] Boddum K., Jensen T. P., Magloire V. (2016). Astrocytic GABA transporter activity modulates excitatory neurotransmission. *Nature Communications*.

[11] Yum D. S., Cho J. H., Choi I. S. (2008). Adenosine A_1_ receptors inhibit GABAergic transmission in rat tuberomammillary nucleus neurons. *Journal of Neurochemistry*.

[12] Alonso-Andres P., Albasanz J. L., Ferrer I., Martin M. (2018). Purine-related metabolites and their converting enzymes are altered in frontal, parietal and temporal cortex at early stages of Alzheimer’s disease pathology. *Brain Pathology*.

[13] Boison D. (2016). Adenosinergic signaling in epilepsy. *Neuropharmacology*.

[14] Melani A., Pantoni L., Corsi C. (1999). Striatal outflow of adenosine, excitatory amino acids, *γ*-aminobutyric acid, and taurine in awake freely moving rats after middle cerebral artery occlusion: correlations with neurological deficit and histopathological damage. *Stroke*.

[15] Navarro G., Borroto-Escuela D. O., Fuxe K., Franco R. (2016). Purinergic signaling in Parkinson’s disease. Relevance for treatment. *Neuropharmacology*.

[16] Angulo E., Casado V., Mallol J. (2003). A_1_ adenosine receptors accumulate in neurodegenerative structures in Alzheimer’s disease and mediate both amyloid precursor protein processing and tau phosphorylation and translocation. *Brain Pathology*.

[17] Horgusluoglu-Moloch E., Nho K., Risacher S. L. (2017). Targeted neurogenesis pathway-based gene analysis identifies ADORA2A associated with hippocampal volume in mild cognitive impairment and Alzheimer’s disease. *Neurobiology of Aging*.

[18] de Castro A. A., da Cunha E. F. F., Pereira A. F. (2018). Insights into the drug repositioning applied to the Alzheimer’s disease treatment and future perspectives. *Current Alzheimer Research*.

[19] Honig L. S., Vellas B., Woodward M. (2018). Trial of solanezumab for mild dementia due to Alzheimer’s disease. *The New England Journal of Medicine*.

[20] Mao J., Huang S., Liu S. (2015). A herbal medicine for Alzheimer’s disease and its active constituents promote neural progenitor proliferation. *Aging Cell*.

[21] Ding Y., Xin C., Zhang C. W. (2018). Natural molecules from Chinese herbs protecting against Parkinson’s disease via anti-oxidative stress. *Frontiers in Aging Neuroscience*.

[22] Ho Y. S., So K. F., Chang R. C. (2011). Drug discovery from Chinese medicine against neurodegeneration in Alzheimer’s and vascular dementia. *Chinese Medicine*.

[23] Zhang Y. L., Liu Y., Kang X. P. (2018). Ginsenoside Rb1 confers neuroprotection via promotion of glutamate transporters in a mouse model of Parkinson’s disease. *Neuropharmacology*.

[24] The PLoS One Staff (2014). Correction: Effects of Huanglian-Jie-Du-Tang and its modified formula on the modulation of amyloid-*β* precursor protein processing in Alzheimer’s disease models. *PLoS One*.

[25] Yu C. J., Zheng M. F., Kuang C. X., Huang W. D., Yang Q. (2010). Oren-gedoku-to and its constituents with therapeutic potential in Alzheimer’s disease inhibit indoleamine 2, 3-dioxygenase activity in vitro. *Journal of Alzheimer's Disease*.

[26] Sun L. M., Zhu B. J., Cao H. T. (2018). Explore the effects of Huang-Lian-Jie-Du-Tang on Alzheimer’s disease by UPLC-QTOF/MS-based plasma metabolomics study. *Journal of Pharmaceutical and Biomedical Analysis*.

[27] Okamoto H., Chino A., Hirasaki Y., Ueda K., Iyo M., Namiki T. (2013). Orengedoku-to augmentation in cases showing partial response to yokukan-san treatment: a case report and literature review of the evidence for use of these Kampo herbal formulae. *Neuropsychiatric Disease and Treatment*.

[28] Chen M., Liao Z., Lu B. (2018). Huang-Lian-Jie-Du-Decoction ameliorates hyperglycemia and insulin resistant in association with gut microbiota modulation. *Frontiers in Microbiology*.

[29] Fa M., Orozco I. J., Francis Y. I., Saeed F., Gong Y., Arancio O. (2010). Preparation of oligomeric *β*-amyloid_1-42_ and induction of synaptic plasticity impairment on hippocampal slices. *Journal of Visualized Experiments*.

[30] Qu S., Meng X., Liu Y., Zhang X., Zhang Y. (2019). Ginsenoside Rb1 prevents MPTP-induced changes in hippocampal memory via regulation of the *α*-synuclein/PSD-95 pathway. *Aging*.

[31] Liu Y., Zong X., Huang J. (2019). Ginsenoside Rb1 regulates prefrontal cortical GABAergic transmission in MPTP-treated mice. *Aging*.

[32] Zhang Y., He X., Meng X. (2017). Regulation of glutamate transporter trafficking by Nedd4-2 in a Parkinson’s disease model. *Cell Death & Disease*.

[33] Lenz E. M., Wilson I. D. (2007). Analytical strategies in metabonomics. *Journal of Proteome Research*.

[34] Morin J. P., Diaz-Cintra S., Bermudez-Rattoni F., Delint-Ramirez I. (2016). Decreased levels of NMDA but not AMPA receptors in the lipid-raft fraction of 3xTg-AD model of Alzheimer’s disease: relation to Arc/Arg3.1 protein expression. *Neurochemistry International*.

[35] Karthick C., Nithiyanandan S., Essa M. M., Guillemin G. J., Jayachandran S. K., Anusuyadevi M. (2018). Time-dependent effect of oligomeric amyloid-*β* (1–42)-induced hippocampal neurodegeneration in rat model of Alzheimer’s disease. *Neurological Research*.

[36] Wang T., Xie X. X., Ji M. (2016). Naturally occurring autoantibodies against A*β* oligomers exhibited more beneficial effects in the treatment of mouse model of Alzheimer’s disease than intravenous immunoglobulin. *Neuropharmacology*.

[37] Kong Y., Ruan L., Qian L., Liu X., Le Y. (2010). Norepinephrine promotes microglia to uptake and degrade amyloid *β* peptide through upregulation of mouse formyl peptide receptor 2 and induction of insulin-degrading enzyme. *The Journal of Neuroscience*.

[38] Palmgren M. G., Nissen P. (2011). P-type ATPases. *Annual Review of Biophysics*.

[39] Garcia D., Shaw R. J. (2017). AMPK: mechanisms of cellular energy sensing and restoration of metabolic balance. *Molecular Cell*.

[40] Herzig S., Shaw R. J. (2018). AMPK: guardian of metabolism and mitochondrial homeostasis. *Nature Reviews Molecular Cell Biology*.

[41] Giau V. V., Wu S. Y., Jamerlan A., An S. S. A., Kim S. Y., Hulme J. (2018). Gut microbiota and their neuroinflammatory implications in Alzheimer’s disease. *Nutrients*.

[42] Bermon S., Petriz B., Kajeniene A., Prestes J., Castell L., Franco O. L. (2015). The microbiota: an exercise immunology perspective. *Exercise Immunology Review*.

[43] Durairajan S. S. K., Iyaswamy A., Shetty S. G. (2017). A modified formulation of Huanglian-Jie-Du-Tang reduces memory impairments and *β*-amyloid plaques in a triple transgenic mouse model of Alzheimer’s disease. *Scientific Reports*.

[44] Xu Y., Zhu N., Xu W. (2018). Inhibition of phosphodiesterase-4 reverses A*β*-induced memory impairment by regulation of HPA axis related cAMP signaling. *Frontiers in Aging Neuroscience*.

[45] Schmid S., Jungwirth B., Gehlert V. (2017). Intracerebroventricular injection of beta-amyloid in mice is associated with long-term cognitive impairment in the modified hole-board test. *Behavioural Brain Research*.

[46] Morroni F., Sita G., Tarozzi A., Rimondini R., Hrelia P. (2016). Early effects of A*β*_1-42_ oligomers injection in mice: involvement of PI3K/Akt/GSK3 and MAPK/ERK1/2 pathways. *Behavioural Brain Research*.

[47] Lu B. L., Li J., Zhou J., Li W. W., Wu H. F. (2016). Tanshinone IIA decreases the levels of inflammation induced by A*β*_1-42_ in brain tissues of Alzheimer’s disease model rats. *Neuroreport*.

[48] Kotani R., Urano Y., Sugimoto H., Noguchi N. (2017). Decrease of amyloid-*β* levels by curcumin derivative via modulation of amyloid-beta protein precursor trafficking. *Journal of Alzheimer's Disease*.

[49] McClure R., Ong H., Janve V. (2017). Aerosol delivery of curcumin reduced amyloid-beta deposition and improved cognitive performance in a transgenic model of Alzheimer’s disease. *Journal of Alzheimer's Disease*.

[50] Liu S. J., Yang C., Zhang Y. (2016). Neuroprotective effect of *β*-asarone against Alzheimer’s disease: regulation of synaptic plasticity by increased expression of SYP and GluR1. *Drug Design, Development and Therapy*.

[51] Wei G., Chen Y. B., Chen D. F. (2013). *β*-Asarone inhibits neuronal apoptosis via the CaMKII/CREB/Bcl-2 signaling pathway in an *in vitro* model and A*β*PP/PS1 mice. *Journal of Alzheimer's Disease*.

[52] Fong S. Y. K., Li C., Ho Y. C. (2017). Brain uptake of bioactive flavones in Scutellariae Radix and its relationship to anxiolytic effect in mice. *Molecular Pharmaceutics*.

[53] Sharma S., Sharma N., Saini A., Nehru B. (2019). Carbenoxolone reverses the amyloid beta 1–42 oligomer–induced oxidative damage and anxiety-related behavior in rats. *Neurotoxicity Research*.

[54] Woods L. T., Ajit D., Camden J. M., Erb L., Weisman G. A. (2016). Purinergic receptors as potential therapeutic targets in Alzheimer’s disease. *Neuropharmacology*.

[55] Silva A. C., Lemos C., Goncalves F. Q. (2018). Blockade of adenosine A_2A_ receptors recovers early deficits of memory and plasticity in the triple transgenic mouse model of Alzheimer’s disease. *Neurobiology of Disease*.

[56] Viana da Silva S., Haberl M. G., Zhang P. (2016). Early synaptic deficits in the APP/PS1 mouse model of Alzheimer’s disease involve neuronal adenosine A_2A_ receptors. *Nature Communications*.

[57] Wardas J. (2002). Neuroprotective role of adenosine in the CNS. *Polish Journal of Pharmacology*.

[58] Zhang C., Rissman R. A., Feng J. (2015). Characterization of ATP alternations in an Alzheimer’s disease transgenic mouse model. *Journal of Alzheimer's Disease*.

[59] Lu Y., Chung H. J., Li Y., Rosenberg P. A. (2003). NMDA receptor-mediated extracellular adenosine accumulation in rat forebrain neurons in culture is associated with inhibition of adenosine kinase. *The European Journal of Neuroscience*.

[60] Lu Y., Rosenberg P. A. (2007). NMDA receptor-mediated extracellular adenosine accumulation is blocked by phosphatase 1/2A inhibitors. *Brain Research*.

[61] Sun X. D., Li L., Liu F. (2016). Lrp4 in astrocytes modulates glutamatergic transmission. *Nature Neuroscience*.

[62] Xiao B., Heath R., Saiu P. (2007). Structural basis for AMP binding to mammalian AMP-activated protein kinase. *Nature*.

[63] Vingtdeux V., Davies P., Dickson D. W., Marambaud P. (2011). AMPK is abnormally activated in tangle- and pre-tangle-bearing neurons in Alzheimer’s disease and other tauopathies. *Acta Neuropathologica*.

[64] Marinangeli C., Didier S., Ahmed T. (2018). AMP-activated protein kinase is essential for the maintenance of energy levels during synaptic activation. *iScience*.

[65] de Vadder F., Mithieux G. (2018). Gut-brain signaling in energy homeostasis: the unexpected role of microbiota-derived succinate. *Journal of Endocrinology*.

[66] Gareau M. G., Wine E., Rodrigues D. M. (2011). Bacterial infection causes stress-induced memory dysfunction in mice. *Gut*.

[67] Little C. S., Hammond C. J., MacIntyre A., Balin B. J., Appelt D. M. (2004). *Chlamydia pneumoniae* induces Alzheimer-like amyloid plaques in brains of BALB/c mice. *Neurobiology of Aging*.

[68] Roubaud-Baudron C., Krolak-Salmon P., Quadrio I., Megraud F., Salles N. (2012). Impact of chronic *Helicobacter pylori* infection on Alzheimer’s disease: preliminary results. *Neurobiology of Aging*.

[69] Zhuang Z. Q., Shen L. L., Li W. W. (2018). Gut microbiota is altered in patients with Alzheimer’s disease. *Journal of Alzheimer's Disease*.

[70] Vogt N. M., Kerby R. L., Dill-McFarland K. A. (2017). Gut microbiome alterations in Alzheimer’s disease. *Scientific Reports*.

[71] Zhao Y., Lukiw W. J. (2018). *Bacteroidetes* neurotoxins and inflammatory neurodegeneration. *Molecular Neurobiology*.

[72] Bauerl C., Collado M. C., Diaz Cuevas A., Vina J., Perez M. G. (2018). Shifts in gut microbiota composition in an APP/PSS1 transgenic mouse model of Alzheimer’s disease during lifespan. *Letters in Applied Microbiology*.

[73] Kong Y., Jiang B., Luo X. (2018). Gut microbiota influences Alzheimer’s disease pathogenesis by regulating acetate in *Drosophila* model. *Future Microbiology*.

[74] Park J. Y., Choi J., Lee Y. (2017). Metagenome analysis of bodily microbiota in a mouse model of Alzheimer disease using bacteria-derived membrane vesicles in blood. *Experimental Neurobiology*.

[75] Zhang L., Wang Y., Xiayu X. (2017). Altered gut microbiota in a mouse model of Alzheimer’s disease. *Journal of Alzheimer's Disease*.

[76] Shen L., Liu L., Ji H. F. (2017). Alzheimer’s disease histological and behavioral manifestations in transgenic mice correlate with specific gut microbiome state. *Journal of Alzheimer's Disease*.

[77] Konikoff T., Gophna U. (2016). *Oscillospira*: a central, enigmatic component of the human gut microbiota. *Trends in Microbiology*.

[78] Kamo T., Akazawa H., Suda W. (2017). Dysbiosis and compositional alterations with aging in the gut microbiota of patients with heart failure. *PLoS One*.

[79] Qian Y., Yang X., Xu S. (2018). Alteration of the fecal microbiota in Chinese patients with Parkinson’s disease. *Brain, Behavior, and Immunity*.

[80] Aguirre M., Bussolo de Souza C., Venema K. (2016). The gut microbiota from lean and obese subjects contribute differently to the fermentation of arabinogalactan and inulin. *PLoS One*.

[81] Zhao R., Liu X., Zhang L., Yang H., Zhang Q. (2019). Current progress of research on neurodegenerative diseases of salvianolic acid B. *Oxidative Medicine and Cellular Longevity*.

[82] Yu H., Yao L., Zhou H. (2014). Neuroprotection against A*β*_25-35_-induced apoptosis by *Salvia miltiorrhiza* extract in SH-SY5Y cells. *Neurochemistry International*.

[83] Huang H. C., Xu K., Jiang Z. F. (2012). Curcumin-mediated neuroprotection against amyloid-*β*-induced mitochondrial dysfunction involves the inhibition of GSK-3*β*. *Journal of Alzheimer's Disease*.

