# Supplementary material for: Modified Huang-Lian-Jie-Du Decoction Ameliorates Aβ Synaptotoxicity in a Murine Model of Alzheimer's Disease
Source: Oxid Med Cell Longev. 2019 Nov 3;2019:8340192. doi: 10.1155/2019/8340192 (PMC6875425; doi:10.1155/2019/8340192)
Supplement: Supplementary Materials — mainly contain the additional behavioral tests, the additional metabolic profiles, and metabolic and microbial differences between modified-HLJDD and HLJDD. [file 8340192.f1.pdf]

Supplementary Data

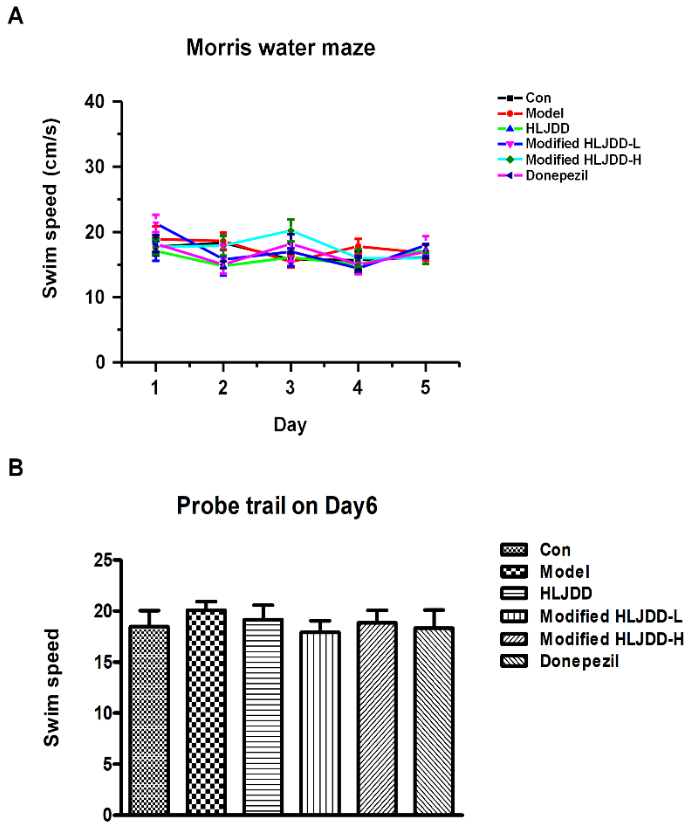

Supplementary Fig. 1 Swim speed in the MWM

(A) Swim speed during a five-day training course in the MWM. (B) Swim speed in the probe trial in the MWM. n = 12 per group. Results are expressed as the mean ± SEM. Statistical significance was determined by one-way ANOVA and Bonferroni tests as *post-hoc* comparisons.

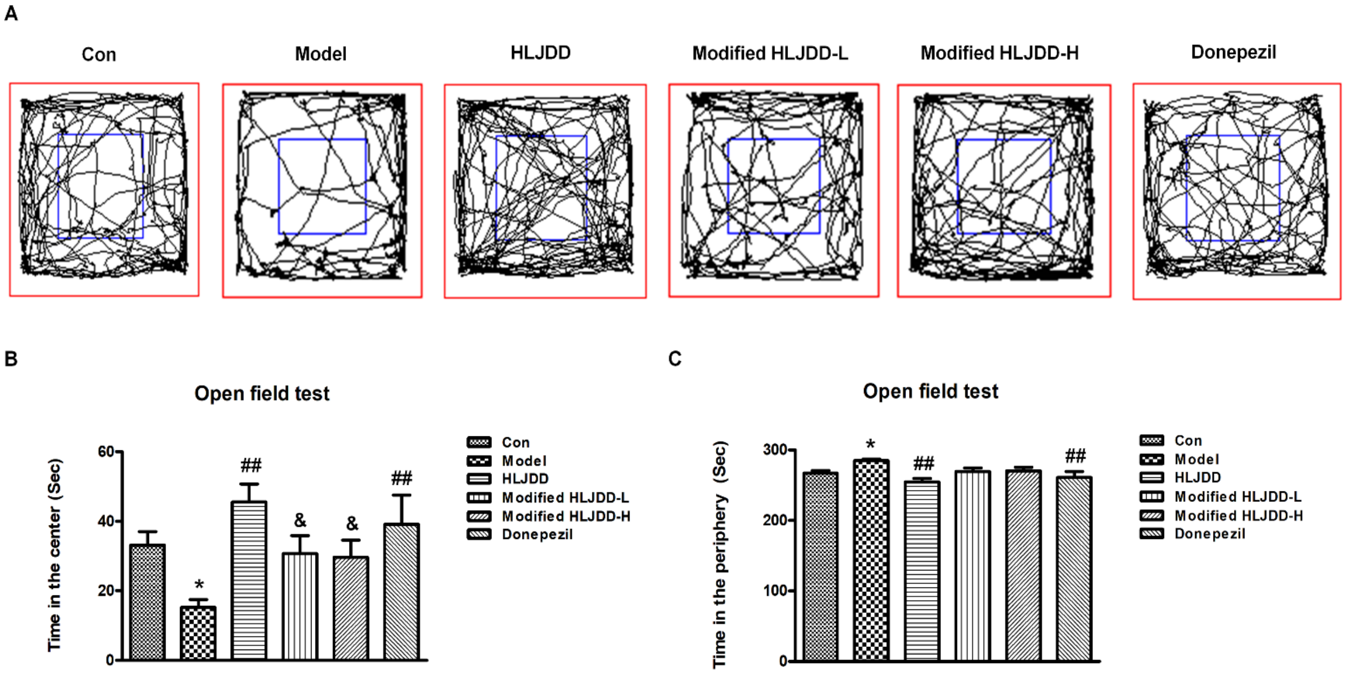

Supplementary Fig. 2 The performance of mice upon modified-HLJDD treatment in the open field test

(A) Representative path tracings in the open field test. (B) Time spent in the center of the open-field after HLJDD, modified-HLJDD-L, modified-HLJDD-H, or Donepezil treatment in the AD model. (C) Time spent in the periphery of the open-field after HLJDD, modified-HLJDD-L, modified-HLJDD-H, or Donepezil treatment in the AD model. n = 12 per group. Results are expressed as the mean ± SEM. $^{*}p < 0.05$ vs. control group; $^{\#\#}p < 0.01$ vs. model group; and $^{\&}p < 0.05$ vs. HLJDD group. Statistical significance was determined by one-way ANOVA and Bonferroni tests as *post-hoc* comparisons.

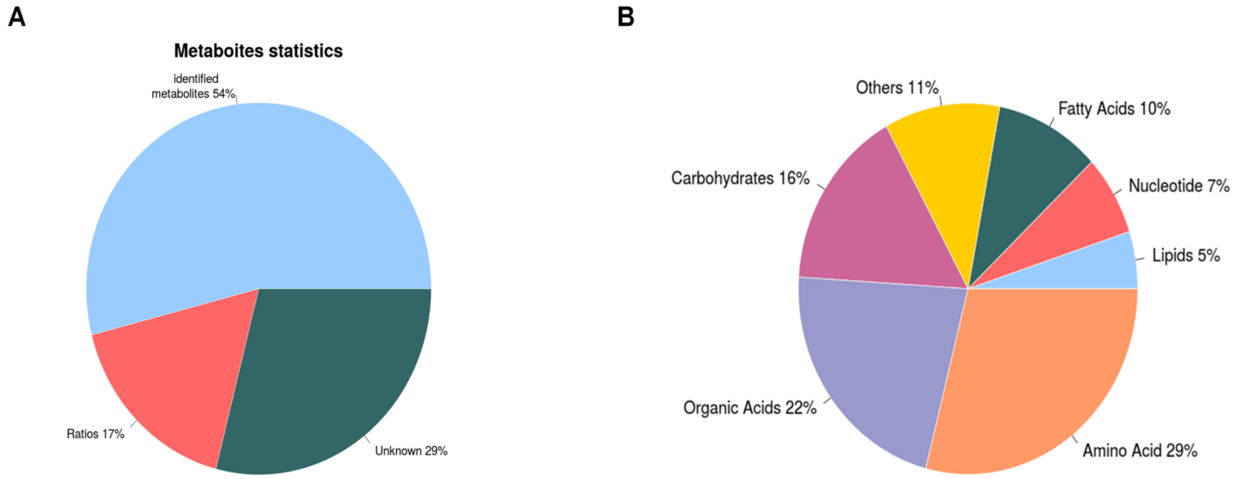

Supplementary Fig. 3 Metabolite classes and compositions detected in the samples

The annotated metabolites and their chemical classes are illustrated in A and B.

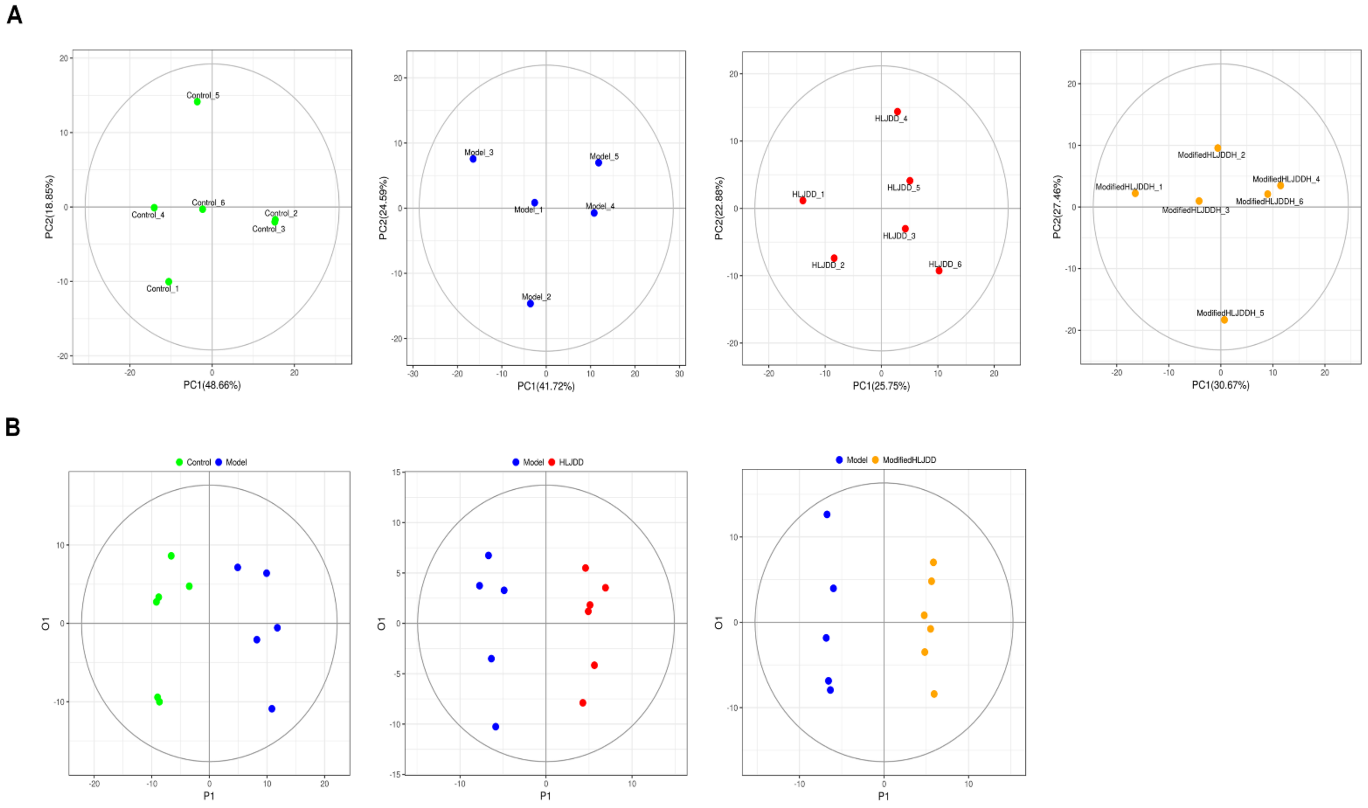

Supplementary Fig. 4 The global metabolic profiles for the subjects from each subgroup

(A) Overview of metabolic profiles of each subgroup using a PCA scores plot. (B) Visualization of overall metabolite profile differences among different groups.

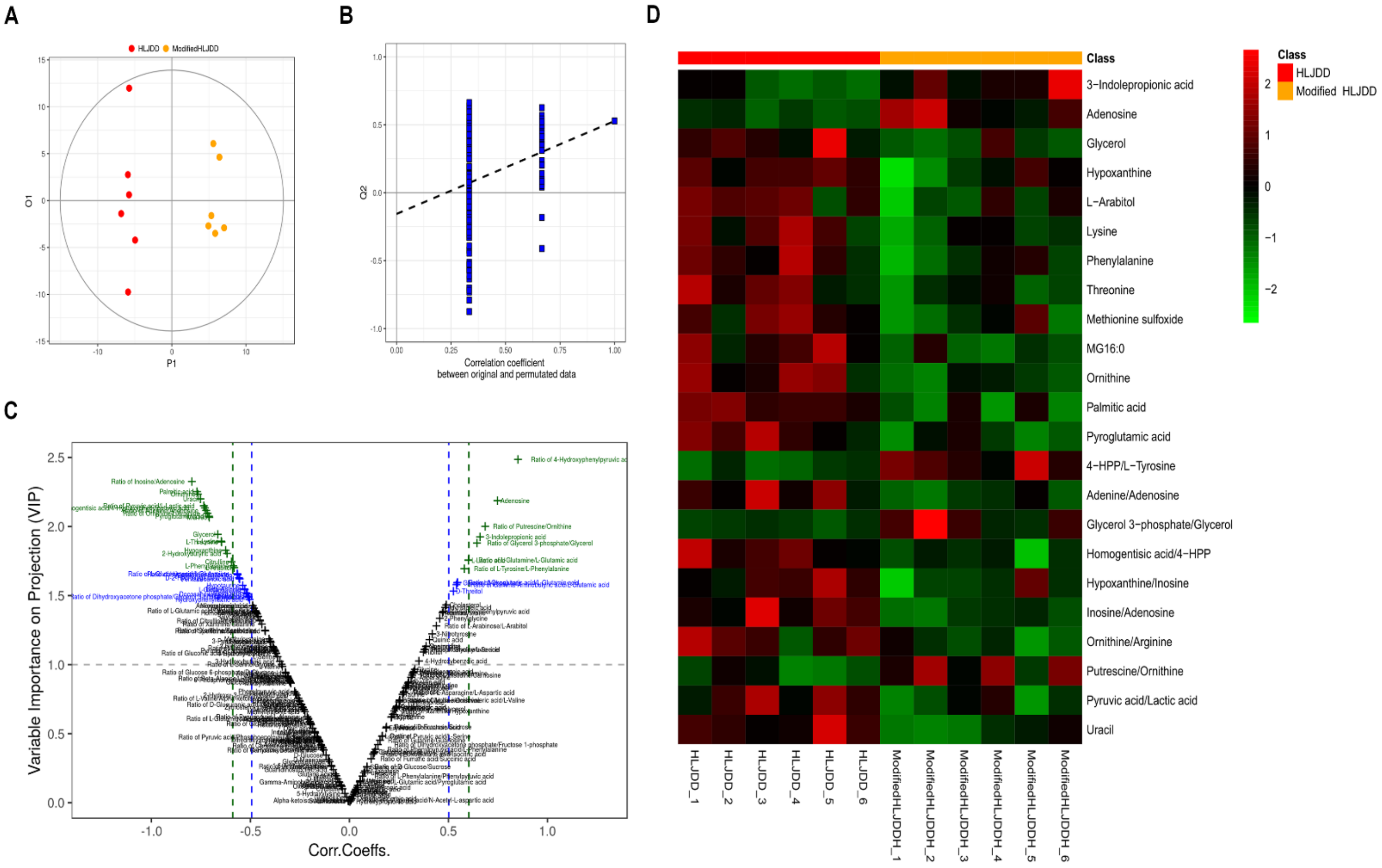

Supplementary Fig. 5 The significant metabolites between HLJDD and modified-HLJDD treatment in the hippocampus in an AD model

The scores plot between HLJDD and modified-HLJDD treatments with an OPLS-DA model and the permutation testing is shown in A and B. (C and D) Volcano plots and heatmaps are used to show the differential metabolites between these two groups. n = 6 per group.

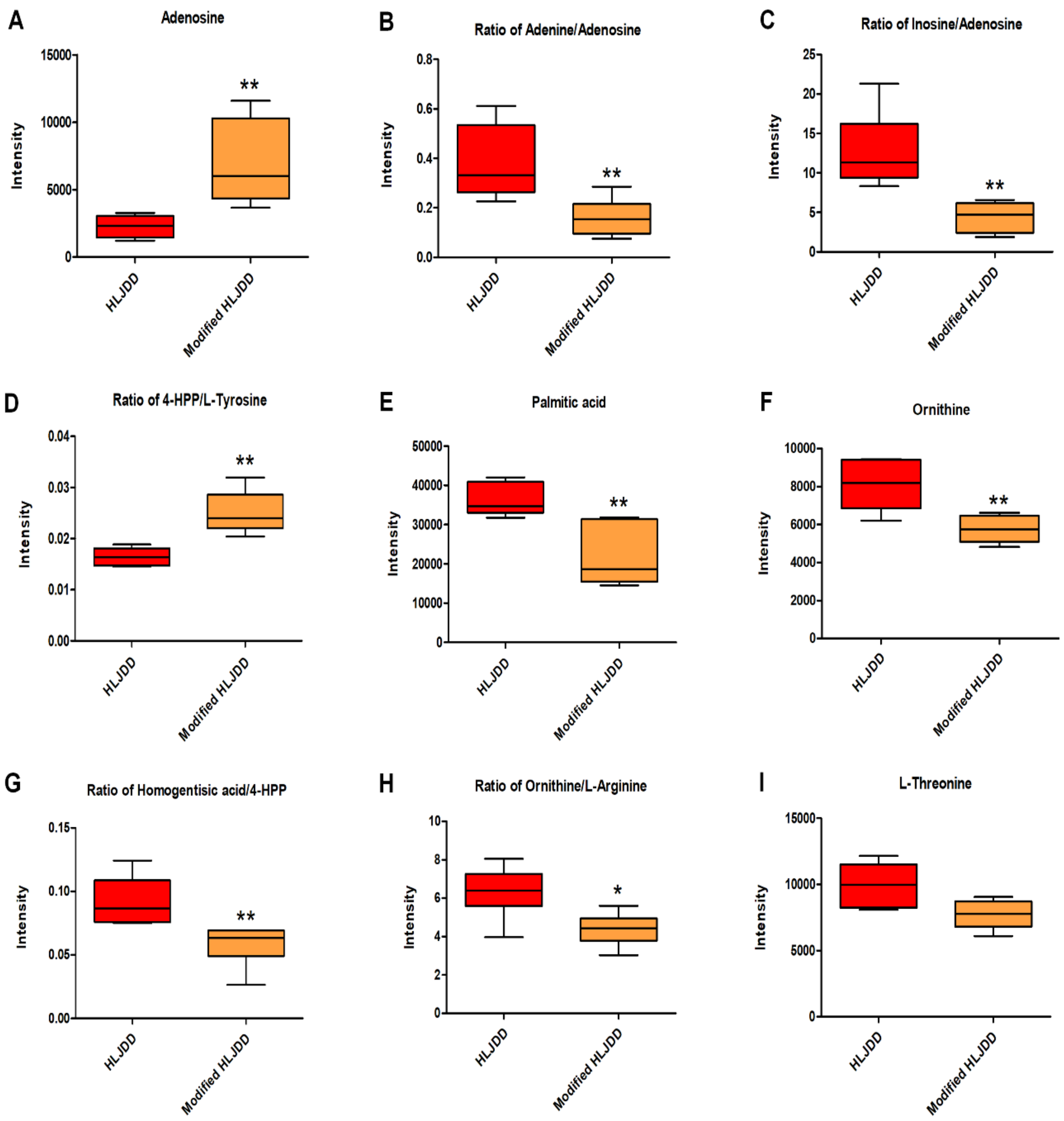

Supplementary Fig. 6 Differential metabolites between HLJDD and modified-HLJDD treatments in the hippocampus in an AD model

(A–I) Top-ranked differential metabolites between the two groups are shown. n = 6 for HLJDD and modified-HLJDD groups. Results are expressed as the mean ± SEM. $^{**}p < 0.01$, $^{*}p < 0.05$. Statistical significance was determined by Student's *t* test.

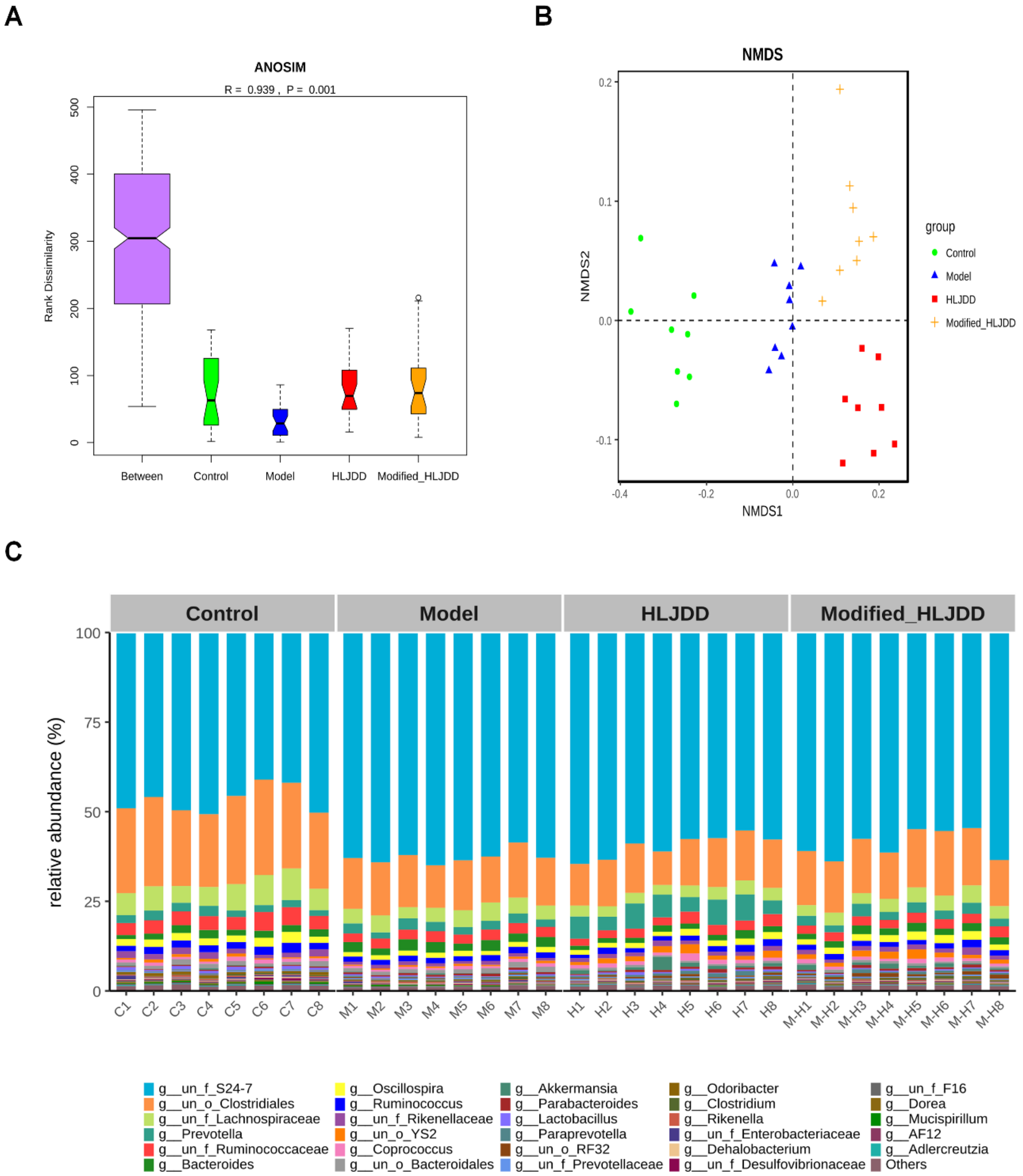

Supplementary Fig. 7 The analysis of sample composition among these four groups

(A–C) ANOSIM, NMDS analysis, and sample species composition analysis among these four groups are shown.

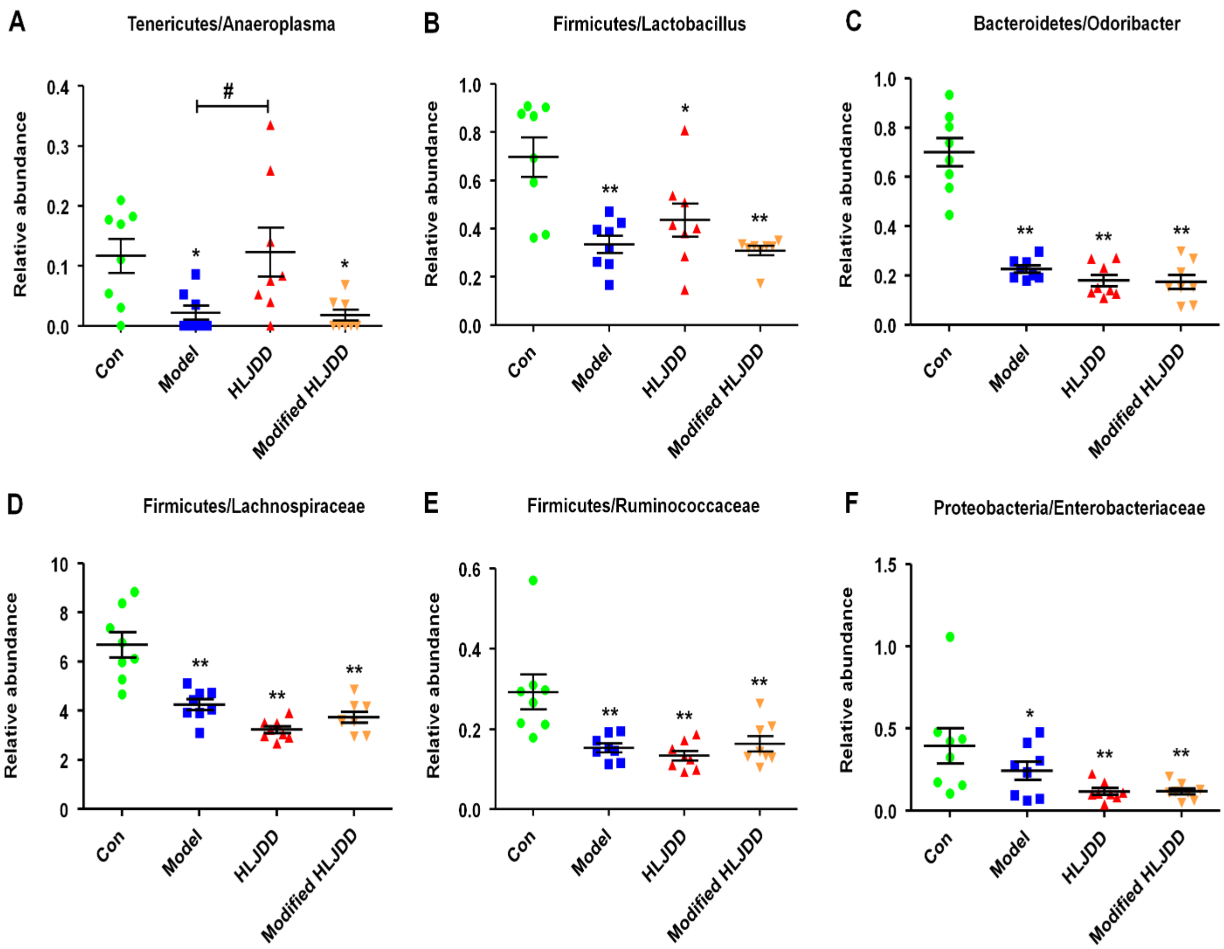

Supplementary Fig. 8 Differential microbiota upon HLJDD and modified-HLJDD treatments in the AD model

(A–I) Differential microbiota among these four groups is shown. n = 8 per group. Results are expressed as the mean ± SEM. $^{**}p < 0.01$, $^{*}p < 0.05$ vs. control group; $^{\#}p < 0.05$ vs. model group. Statistical significance was determined by one-way ANOVA and Bonferroni tests as *post-hoc* comparisons.

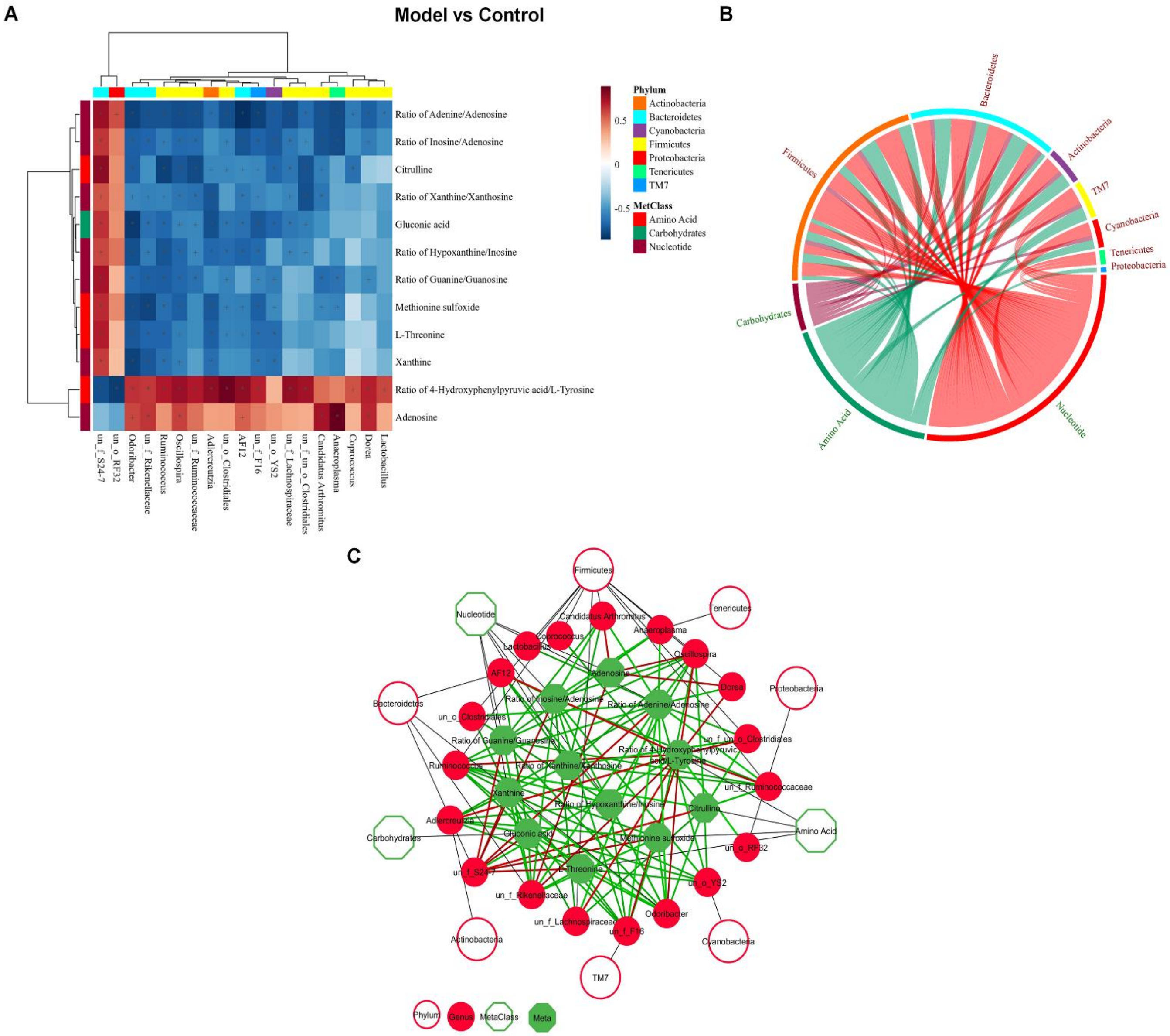

Supplementary Fig. 9 The correlation between significant hippocampal metabolites and gut microbiota

(A–C) The correlation between significant hippocampal metabolites and gut microbiota is shown for AD model vs. control in the form of a heatmap, chord diagram, and network diagram.

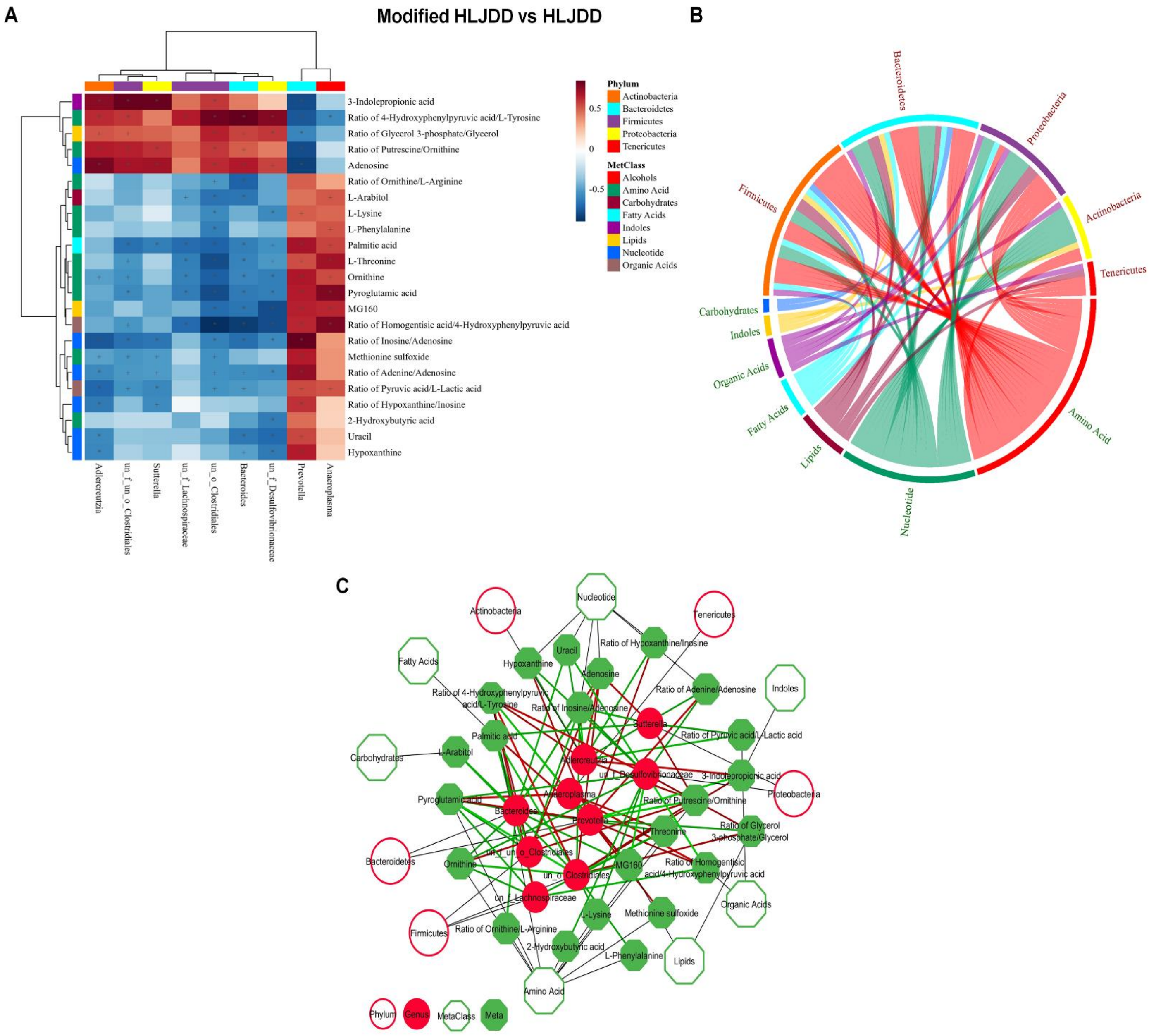

Supplementary Fig. 10 The correlation between significant hippocampal metabolites and gut microbiota

(A–C) The correlation between significant hippocampal metabolites and gut microbiota is shown for modified-HLJDD vs. HLJDD in the form of a heatmap, chord diagram, and network diagram.
